# Haemopedia RNA-seq: a database of gene expression during haematopoiesis in mice and humans

**DOI:** 10.1093/nar/gky1020

**Published:** 2018-11-05

**Authors:** Jarny Choi, Tracey M Baldwin, Mae Wong, Jessica E Bolden, Kirsten A Fairfax, Erin C Lucas, Rebecca Cole, Christine Biben, Clare Morgan, Kerry A Ramsay, Ashley P Ng, Maria Kauppi, Lynn M Corcoran, Wei Shi, Nick Wilson, Michael J Wilson, Warren S Alexander, Douglas J Hilton, Carolyn A de Graaf

**Affiliations:** 1 Molecular Medicine Division, The Walter and Eliza Hall Institute of Medical Research, Parkville, Victoria, Australia; 2 Centre for Stem Cell Systems, Anatomy and Neuroscience Department, The University of Melbourne, Parkville, Victoria, Australia; 3 CSL Limited, Parkville, Victoria, Australia; 4 Department of Medical Biology, The University of Melbourne, Parkville, Victoria, Australia; 5 Cancer and Haematology Division, The Walter and Eliza Hall Institute of Medical Research, Parkville, Victoria, Australia; 6 Molecular Immunology Division, The Walter and Eliza Hall Institute of Medical Research, Parkville, Victoria, Australia; 7 Bioinformatics Division, The Walter and Eliza Hall Institute of Medical Research, Parkville, Victoria, Australia; 8 Department of Computing and Information Systems, The University of Melbourne, Parkville, Victoria, Australia

## Abstract

During haematopoiesis, haematopoietic stem cells differentiate into restricted potential progenitors before maturing into the many lineages required for oxygen transport, wound healing and immune response. We have updated Haemopedia, a database of gene-expression profiles from a broad spectrum of haematopoietic cells, to include RNA-seq gene-expression data from both mice and humans. The Haemopedia RNA-seq data set covers a wide range of lineages and progenitors, with 57 mouse blood cell types (flow sorted populations from healthy mice) and 12 human blood cell types. This data set has been made accessible for exploration and analysis, to researchers and clinicians with limited bioinformatics experience, on our online portal Haemosphere: https://www.haemosphere.org. Haemosphere also includes nine other publicly available high-quality data sets relevant to haematopoiesis. We have added the ability to compare gene expression across data sets and species by curating data sets with shared lineage designations or to view expression gene vs gene, with all plots available for download by the user.

## INTRODUCTION

Haematopoiesis is the process that forms the cells of the blood; haematopoietic cells range from stem cells that are capable of self renewal and of reconstituting all other haematopoietic lineages, to the many mature cells that fight infection, carry oxygen and clot the blood. Each of the cell types can be distinguished on the basis of cell surface markers and has a unique signature of gene expression that enables these cells to carry out their diverse functions. The lineages and the molecular pathways of the human and mouse haematopoietic systems are evolutionarily conserved, and this conservation has been used to gain insight into the molecular basis of blood cell production and human disease.

We have previously published a comprehensive database of microarray gene expression profiles from FACS sorted samples of wildtype mouse haematopoietic cell types, Haemopedia (1), with associated analysis and data visualisation tools on www.haemosphere.org, which has been a useful and popular resource for researchers working in haematopoiesis and cellular differentiation.

Here we present an improved database, Haemopedia RNA-seq, that contains high quality RNA-seq gene-expression data that covers progenitors and all the major mature haematopoietic lineages in mice and 7 mature lineages in humans, allowing for comparison of expression between mice and humans. The increased sensitivity of the RNA-seq platform avoids confounding factors such as probe design, so can detect a wider variety of transcripts and isoforms than microarrays. It also allows for more accurate detection calls for lowly expressed genes, which can be particularly important for some key drivers of haematopoiesis like transcription factors and receptors where low expression may be sufficient for biological activity.

While there are other online databases available that contain gene expression data covering a range of haematopoietic cell types, such as ImmGen (2), BloodSpot (3), Differentiation Map (4), GeneExpression Commons (5), Haematopoietic Fingerprints (6), Blueprint (7), they have limited lineage coverage, are made up of microarray data or have user interfaces that are limited to select data sets or features.

The Haemopedia RNA-seq data sets are available via our web portal, Haemosphere (www.haemosphere.org), which is intuitive for non-bioinformaticians to use. We have improved the analytical functionality of Haemosphere by allowing the user to identify genes which are highly expressed in a single lineage or cell type relative to other cell types within the atlas, and by adding new data visualizations for comparing genes across data sets and species and comparing the expression of two genes. These complement the core functions of Haemosphere which are to provide expression plots of genes across the multiple lineages and differentiation stages of haematopoiesis and to allow the user to perform differential gene expression analyses corrected for multiple testing. The gene sets and plots generated by the user are available for download.

## HAEMOPEDIA DATA CONTENT UPDATES

### Sample collection

**Figure 1.**
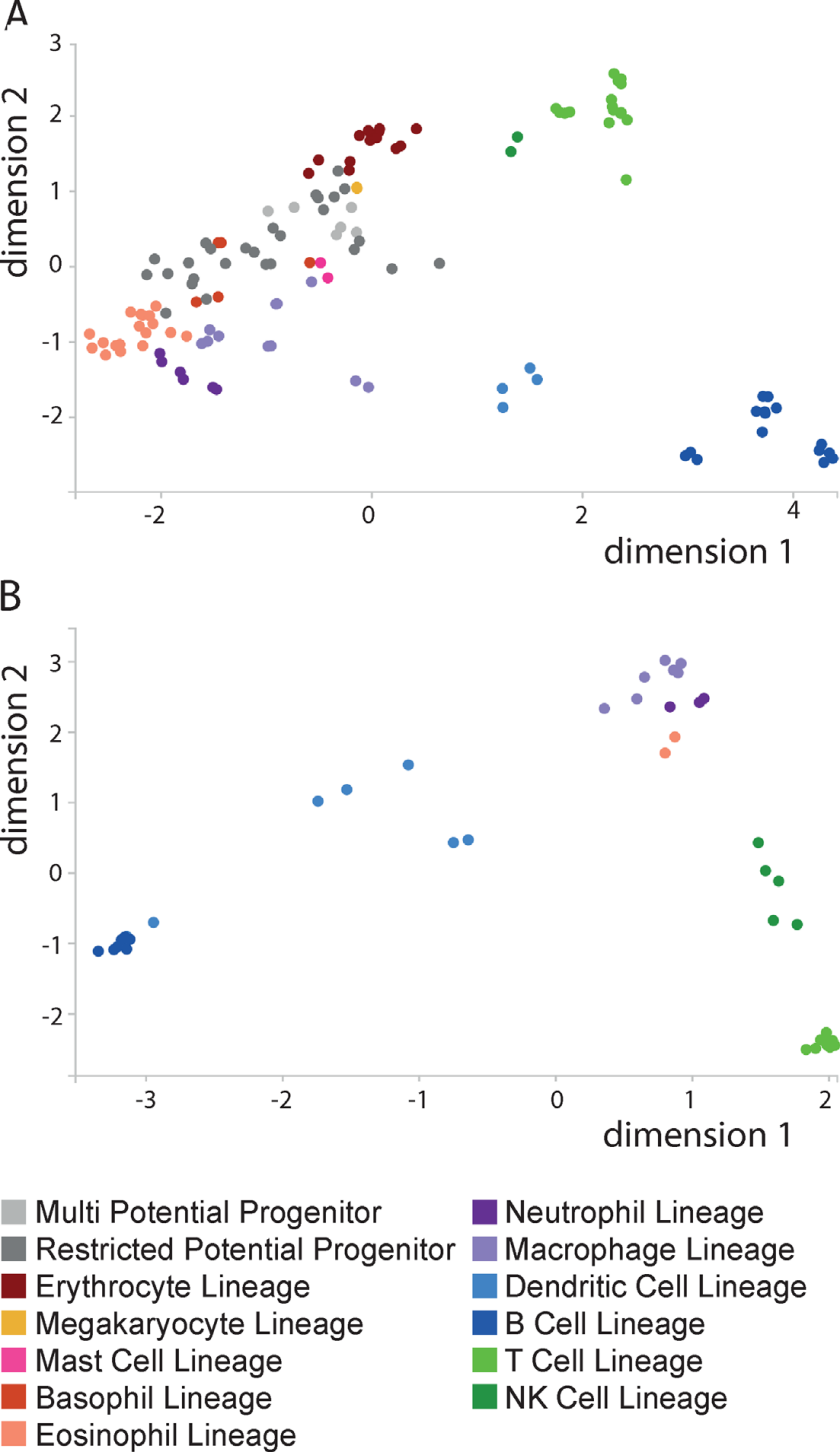
Multi-dimensional scaling plot. Multi-dimensional scaling (MDS) plots of the (A) Haemopedia mouse RNA-seq and (B) Haemopedia human RNA-seq data sets. Plots were produced by Haemosphere, using the Euclidean distance between the 500 most variable genes in each data set, with the dimension reduction performed with principal coordinates analysis. Each dot represents a sample and they are coloured by lineage as shown in the key.

The Haemopedia RNA-seq data sets contains transcriptional profiles for 171 haematopoietic samples from both mouse and human (Table 1). As shown on a multi-dimensional scaling plot produced in Haemosphere, the Haemopedia RNA-seq data set samples cluster according to cell lineage (Figure 1). The multi-dimensional scaling plot can be accessed by selecting any data set from the ‘Data Sets’ page.

**Table 1.** Summary of RNA-seq data sets included in the repository

Data set	Species	#Samp	MP	RP	Er	Mk	Ms	Ba	Eo	Nt	Mc	DC	B	T	IL	Ref
Haemopedia-RNAseq-Mouse	Mouse	129	3	11	6	1	1	3	6	2	5	2	8	8	1	
Haemopedia-RNAseq-Human	Human	42							1	1	2	3	2	2	1	
ImmGen-RNASeq	Mouse	157	6				1			3	4	3	18	32	11	(2)
Linsley *et al.*	Human	114								1	1		1	2	1	(13)

#Samp = number of samples in data set. The lineage columns give the number of different cell types of that lineage included in the data set. MP = multipotential progenitor, RP = restricted potential progenitor, Er = erythrocyte lineage, Mk = megakaryocyte lineage, Ms = mast cell lineage, Ba = basophil lineage, Eo = eosinophil lineage, Nt = neutrophil lineage, Mc = macrophage lineage, DC = dendritic cell lineage, B = B cell lineage, T = T cell lineage, IL = innate lymphocyte lineage, Ref = publication reference.

The Haemopedia mouse RNA-seq samples cover 57 haematopoietic cell types from all major lineages, including a comprehensive number of myeloid progenitors and mature granulocytes (Table 1). All murine samples were collected on a C57BL/6 background and sorted by flow cytometry (immunophenotypes shown in Supplementary Table S1 with antibodies listed in Supplementary Table S2 and detailed methods in supplementary methods). IRF8-GFP (8) and Blimp-GFP (9) mice were used to identify certain populations as in Supplementary Table S2. All procedures involving animals were approved by The Walter and Eliza Hall Institute of Medical Research Animal Ethics Committee. Some samples included in this data set have been previously published, as indicated in Supplementary Table S1 (10–12).

The Haemopedia Human RNA-seq samples represent 12 cell types across 7 lineages, and were collected according to the immunophenotypes shown in Supplementary Table S3, with antibodies as given in Supplementary Table S2 and the detailed methods in supplementary methods. They were collected from buffy packs from anonymous healthy donors, with donor number as indicated in Supplementary Table S3. The collection of human buffy coats was approved by the Blood Service Human Research Ethics Committee (Australian Red Cross) and governed by the material supply agreement between CSL Ltd and the Australian Red Cross Blood Service. Written informed consent was obtained from all volunteer donors recruited to the study.

### Sample processing

The RNA extraction protocol is detailed in the supplementary methods. Overall, 100–200 ng total RNA per sample was submitted to the Australian Genome Research Facility for high throughput mRNA-sequencing. Libraries (mRNA) were synthesized using Illumina's TruSeq Stranded mRNA protocol, and 100 bp reads generated with an Illumina HiSeq 2500 (Illumina). Samples were aligned with Rsubread (13) to either the mouse genome GRCm38 or the human genome GRCh38, both Ensembl release 84. Reads were assigned to genes with featureCounts (14). A summary of read mapping and quantification results can be found in Supplementary Tables S1 and S3. Species orthologues were mapped with the Mouse Genome Informatics Homology report (informatics.jax.org/downloads/reports/index.html#homology).

The human GRCh38 genome covers 60 504 genes, with 19 901 of them expressed at counts per million (cpm) >1 in 2 or more samples in the Haemopedia Human RNA-seq data set. The mouse GRCm38 annotation covers 47 643 genes. In our Haemopedia mouse RNA-seq data set, 25 957 genes were expressed at counts per million >1 in 2 or more samples. This represents a significant improvement compared to the annotation for the Illumina MouseWG-6 V 2.0 BeadChips used in the original Haemopedia data set that had 34 031 probes covering 19 699 genes.

### Comparison data sets

We have included two other recent haematopoietic RNA-seq data sets in Haemosphere – Immgen (2) and Linsley *et al.* (15). A summary of the cell types represented in these data sets is given in Table 1. There are also 8 microarray data sets available in Haemosphere (1,2,4–6,16–18), including the original Haemopedia data set. These are summarized in Supplementary Table S4.

## FUNCTIONALITY UPDATES

### Data set comparison

**Figure 2.**
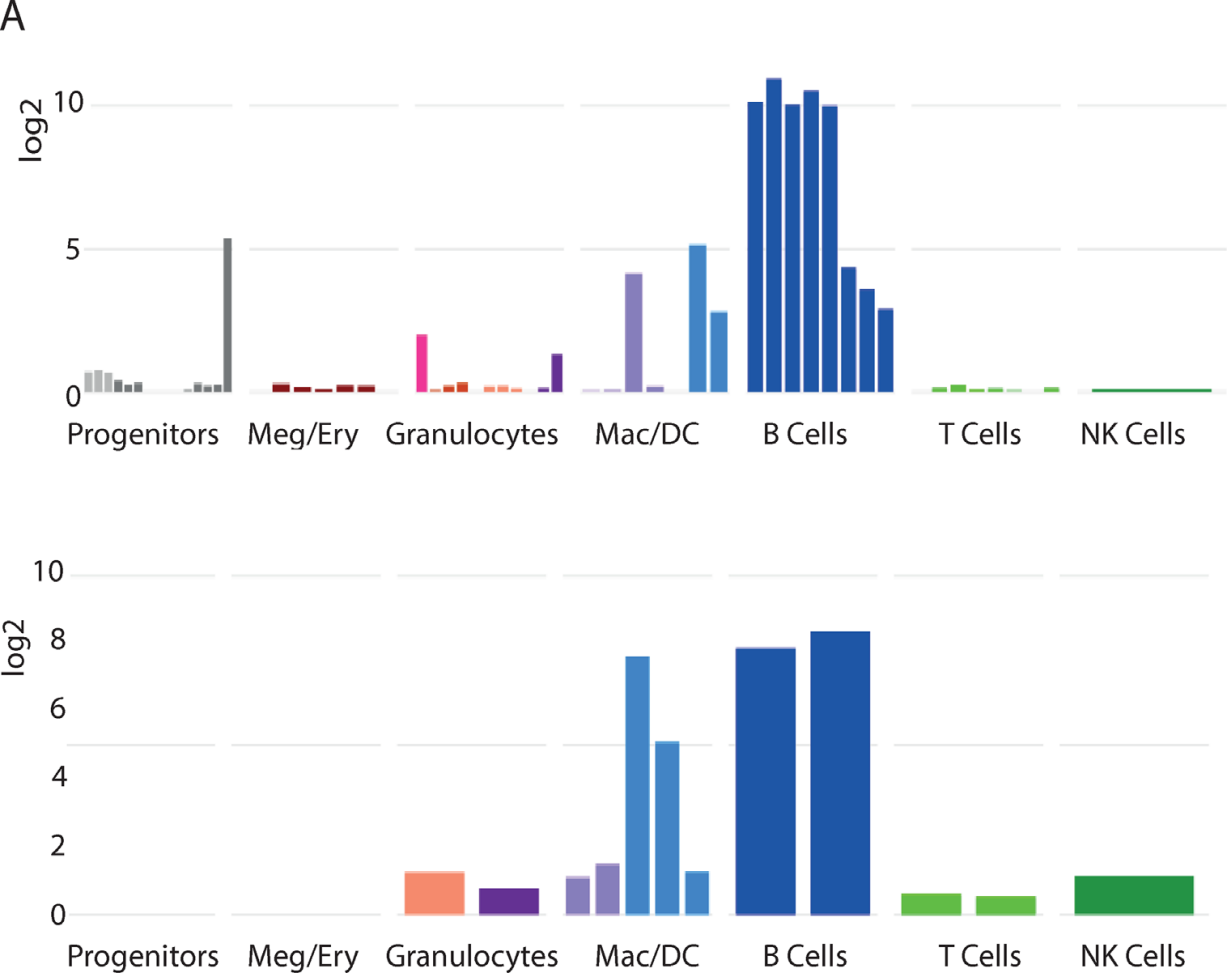
Multi-species plot. Example multi-species graphs from Haemosphere comparing *Cd19* expression in the Haemopedia mouse RNA-seq data set to its orthologue *CD19* in the Haemopedia human RNA-seq data set. Cell types have been assigned to lineages, and plotted with shared colours according to the colour scheme in Figure 1. Some related lineages, such as granulocytes, have been grouped together to simplify the view. On the website, hovering over each bar reveals the cell type name and clicking on the data set name takes the user to the gene's expression plot in that data set to display more detail.

The 12 data sets that we have included in Haemosphere each contain a different set of cell types, even when only comparing within the one species. However, the expression of many genes is correlated across lineages in different species. In order to compare genes across species and data sets, we have manually curated the cell types in each data set with a common set of lineage annotations. We have used this to produce a multi-species plot, where the expression pattern of a gene can be compared between mouse and human (Figure 2), or more generally between data sets, which also allows cross-platform comparison between the RNA-seq and microarray data sets. This plot can be accessed from a gene's expression profile page or from gene lists where ‘multi-species’ can be selected from the Expression column.

### Gene correlation

Genes that operate in a common pathway often display correlated expression profiles. To aid the user in comparing gene expression profiles, we have created an interactive ‘Gene Vs Gene’ plot, allowing the user to compare the expression of two genes across each cell type, coloured by lineage. It can be selected from any gene expression profile or gene list.

**Figure 3.**
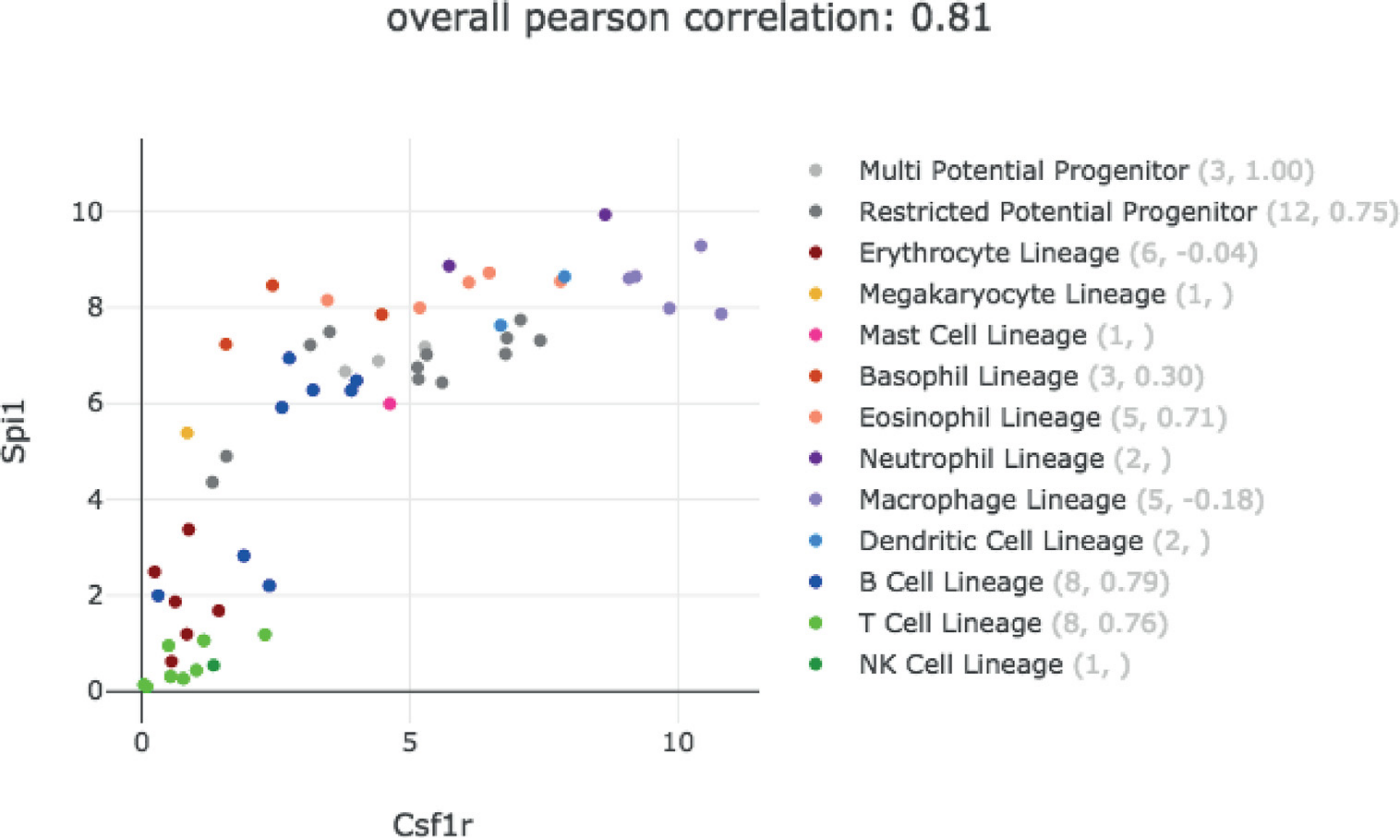
Gene versus gene plot. Example of gene versus gene plot from Haemosphere using the Haemopedia mouse RNA-seq data set. The expression of the transcription factor *Spi1* is plotted against *Csf1r* expression. Each dot represents average expression for a different cell type and is coloured according to lineage. The number of cell types in a lineage and the Pearson correlation within the lineage is shown in brackets in the legend.

In Figure 3 we show the Gene vs Gene plot taken from Haemosphere for the Haemopedia mouse RNA-seq data set for the transcription factor *Spi1* (*Pu1*), which has been shown to be critical in the regulation of myeloid commitment (19), and its target, colony stimulating factor 1 receptor (*Csf1r*) (20). The expression of these two genes is highly correlated (Pearson correlation 0.77). The plot also displays their degree of correlation in each lineage, showing that, for example, in Restricted Potential Progenitors their correlation is 0.82. To facilitate interpretation, cell labels appear when the user hovers over them, and the graph can be downloaded.

The gene versus gene plot complements our ‘Find Similar’ function (1), which returns the 100 most positively and negatively correlated genes to a gene of interest and can be selected from a gene's expression profile page.

### Selection of differentially expressed genes

We have made it simple for users without extensive bioinformatics experience to identify genes that are differentially expressed between two cell types or lineages. This is available on Haemosphere with differential expression analyses on RNA-seq data powered by EdgeR (21) and Voom (22) using ‘Differential Expression Search’ under the ‘Searches’ subheading. Here the user can select the data set of interest, how they wish to group the cells (such as by lineage or cell type) and groups they want to compare. A table of differentially expressed genes is returned with the log fold change and adjusted *P* value. This table can then be downloaded for further analyses, viewed as a heatmap across all cell types in the data set for the top 300 genes or mapped to the mouse or human orthologues. To facilitate customised analyses for advanced users, in addition to the raw data set files, the R code used to generate the query, as well as the relevant R objects, are all available to download.

**Figure 4.**
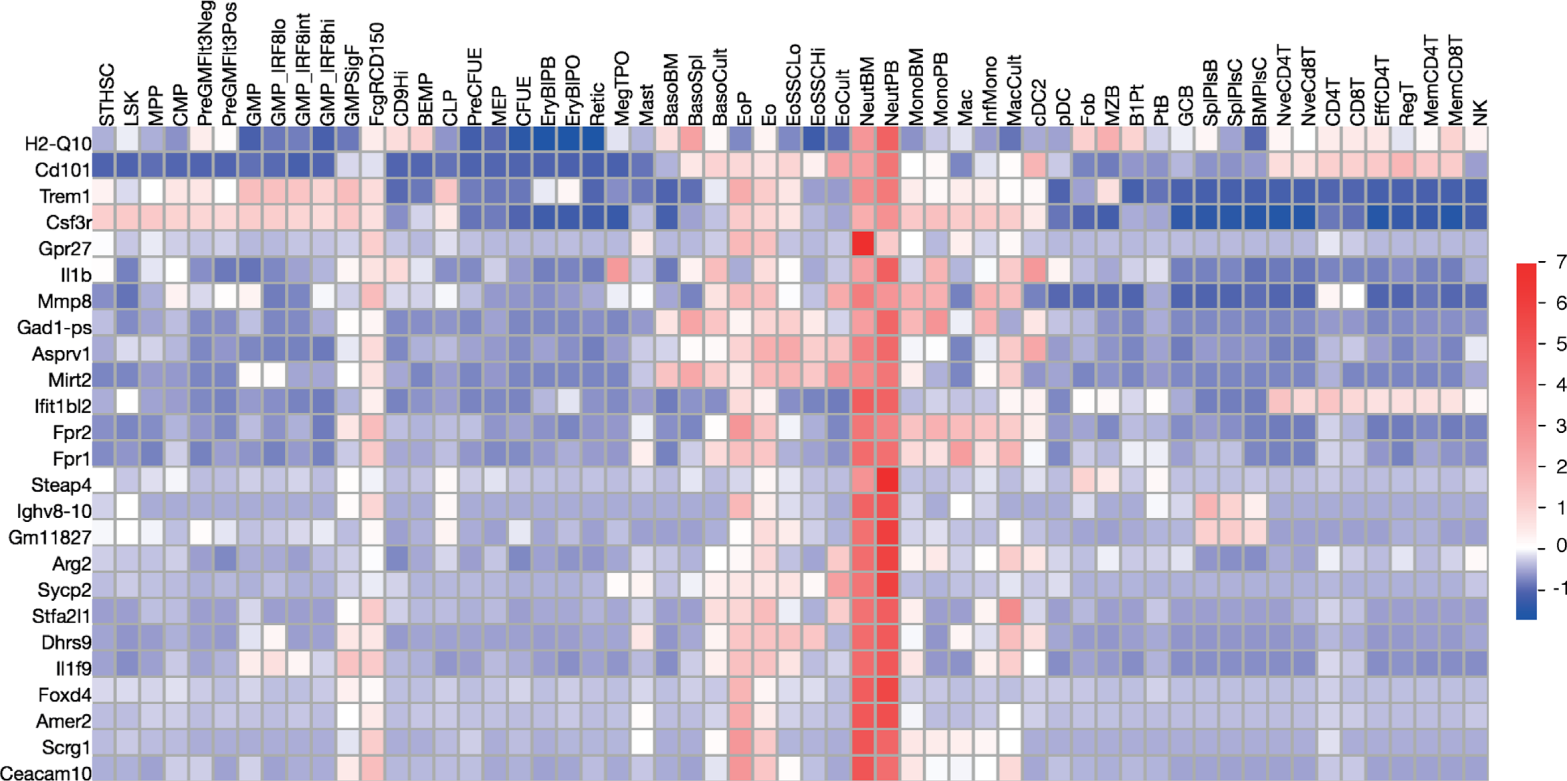
Heatmap of genes with high expression in neutrophils. This heatmap was generated from haemosphere.org using the Haemopedia mouse RNA-seq data set and shows the 25 most enriched genes in the neutrophil lineage as selected by Haemosphere's ‘High Expression Search’ function. Squares are coloured by row based *z* score.

Drug discovery or scientific manipulation of cells means that that there is often interest in finding genes that are specifically expressed in a particular cell type or lineage. This requires more than just pairwise expression comparison. We have therefore created a ‘High Expression Search’ (also accessible under the ‘Searches’ subheading) to allow for multiple pairwise comparisons rather than just comparing between two groups. The user can choose the data set and select a single group for which the multiple pairwise comparisons can be undertaken. The algorithm selects genes that have their highest expression in the selected sample group. Each gene is scored for log_2_ fold change between the average of the sample group and the highest average value of the other sample groups, and only genes with positive scores are returned. The gene set can then be used for other functions, including creation of a heatmap. The Haemosphere heatmap can be accessed from any page with a gene set by clicking on the heatmap icon. A Haemosphere heatmap showing the top 25 genes from a neutrophil lineage High Expression search in the Haemopedia mouse RNA-seq data set is shown Figure 4.

### Functionality overview

To guide users through the major functions of the database, we have included tours of main pages which can be accessed by clicking on the ‘?’ symbol on the pages.

### Data accessibility

All data and analyses are freely available at haemosphere.org without registration or a login. An option to register is provided as an additional feature, so that users can save their gene sets or create custom subsets of larger data sets.

Counts, TPMs (transcripts per kilobase million) and sample information for the Haemopedia RNA-seq data sets and all other data sets are available for download from the ‘Data Sets’ page on haemosphere.org. The raw RNA-seq data have been deposited in NCBI’s Gene Expression Omnibus (23). The human data is accessible through GEO Series accession number GSE115736 and the mouse through accession number GSE116177.

Haemosphere is a python pyramid (https://trypyramid.com/) application, using mainly HDF5 (https://portal.hdfgroup.org) format for data. The code repository will become public in near future. Its design and structure is based on BioPyramid (24), which is freely available at http://github.com/jarny/biopyramid.

## CONCLUSION

Here we have presented a web-based data portal allowing comparison of many human and mouse haematopoietic cell types in diverse lineages using high quality RNA-seq data sets. We have provided tools to allow bench scientists and clinicians to easily access and mine this gene-expression data, with a consistent lineage annotation to make the website easy to navigate. Users are able to export relevant figures and gene sets. Our platform also allows the integration of additional RNA-seq samples as they become available, to provide an even more comprehensive insight into gene-expression during haematopoiesis.

Users can identify genes that are correlated, differentially expressed between cell types or highly expressed in a particular lineage, aiding in the identification of potential therapeutic targets and novel co-regulated genes. The ease of comparison between mouse and human data sets supports the translation of experimental findings from animal models into the clinic.

## DATA AVAILABILITY

The Haemopedia RNA-seq data sets are available on the Haemosphere platform at https://www.haemosphere.org.

The raw RNA-seq data have been deposited in NCBI’s Gene Expression Omnibus (23). The human data is accessible through GEO Series accession number GSE115736 and the mouse through accession number GSE116177.

## Supplementary Material

Supplementary Data
