# Supplementary material for: Haemopedia RNA-seq: a database of gene expression during haematopoiesis in mice and humans
Source: Nucleic Acids Res. 2018 Nov 5;47(Database issue):D780–5. doi: 10.1093/nar/gky1020 (PMC6324085; doi:10.1093/nar/gky1020)
Supplement: Supplementary Data [file gky1020_supplemental_files.zip › Haemopedia Supp Meth and Legends.docx]

Supplementary Methods

**Flow cytometry – gating strategy and sorting of mouse cell subsets**

Bone marrow, spleens, peripheral blood, thymus and peritoneal cavity lavage cells were collected from 7-12 week old mice. All replicates were from different individuals, but in rare populations tissues from several mice were pooled for a single sample. Peripheral blood was collected from the retro-orbital sinus into Microtainer tubes containing EDTA (BD Biosciences). Single cell suspensions of spleen and thymus cells were prepared using a 100 um cell strainer (BD Falcon). Enrichment of cells of interest was performed as noted in the method column in Supplementary Table 1. Cells were stained with cocktails of antibodies as indicated Supplementary Tables 1 and 2. Cells were resuspended in PBS/2%FCS, 2 mM EDTA, 1 mg/mL Propidium Iodide (Sigma) to enable identification and exclusion of dead cells.

For production of cultured eosinophils and macrophages (eoCult and eoMac populations), fetal livers were extracted from day 14 embryos and following Ter119 depletion were cultured for 3 days in IMDM (Gibco) supplemented with 1% penicillin/streptomycin, 15% FBS (SAFC), 100 ng/ml SCF (PeproTech), 10 ng/ml IL-­6 (produced in-house), 50 ng/ml TPO (produced in-house) and 5ng/ml Flt3 (produced in-house). For production of eosinophils (eoCult population), cells were washed and cultured for a further 10 days in IMDM with 10% FBS and 5 ng/ml IL-5 (PeproTech) at 37°C in 10% CO2. Cells were then stained for Siglec-F expression and sorted for Siglec-F+ cells on a flow cytometer. Macrophages (macCult population) were produced by culturing for a further 5 days in 10ng/ml MCSF (produced in-house) in IMDM with 10% FBS at 37°C in 10% CO2, and were stained for Mac1 and F4/80 expression and sorted for double positive cells on a flow cytometer.

Megakaryocytes were derived from bone marrow cultures. Bone marrow was red cell lysed then stained with lineage cocktail (IL7ra, Gr1 Ter119 CD3 B220), Sca1, cKit and CD150 antibodies. The Lin- Sca1- cKit+ CD150+ fraction was collected on a flow cytometer. Cells were then cultured for 3 days in (37°C 10% CO2) in StemPro serum-free medium with the nutrient supplement provided (ThermoFisher #10639011) and 100 ng/ml TPO (produced in house).

# For the majority of populations, FACS plots of the gating strategy used are shown in Supplementary Figure 1. Otherwise, a reference to a published FACS gating profile is given in Supplementary Table 1. Purity checks were performed by FACS reanalysis where feasible, with 94 +/- 5 (mean +/- sd) purity.

# Flow cytometry- gating strategy and sorting of human cell subsets

Healthy donor (de-identified) buffy coats were obtained from the Australian Red Cross (Melbourne, Australia). Peripheral blood mononuclear cells (PBMCs) and granulocytes were isolated using Ficoll-Paque Plus (#17-1440-03, GE Healthcare), as per manufacturer’s instructions. The following methods describe the gating strategy for isolation of human cell subsets on the flow cytometer (FACS Aria 1, BD). Debris, doublets and dead cells (Propidium Iodide+ (Miltenyi Biotec)) were excluded from collection. For some cell subsets, magnetic bead separation techniques (positive and negative selection) were used and incorporated the Automacs Pro Separator (Miltenyi Biotec). Data were analyzed using Flowjo v8.

2x10^8^ PBMCs were stained for antibodies against CD3, CD4, CD8 CD56, CD19 and CD27 antigens. From the CD3+ CD19- CD56- sub-population, CD8 T-cells (CD8+) and CD4 T-cells (CD4+) were identified. From the CD3- cells, B-cells were identified as CD19+ CD56- with further differentiation to naïve B-cells (CD27-) and memory B-cells (CD27+). From the CD3- CD19- sub-population, NK cells (CD56+) were identified.

In a separate PBMC sort from the same donor, CD3+ T-cells were depleted from 4x10^8^ PBMCs by positive selection magnetic bead separation, using the CD3 microbeads (#130-050-101, Miltenyi Biotec). The remaining 10^8^ PBMCs were stained with antibodies against CD14, BDCA1, BDCA2 and CD123 antigens. CD14hi BDCA2- cells distinguished the monocytes which were also BDCA1-and CD123lo. CD14int BDCA2- CD123int BDCA1- cells were termed non-classical monocytes. From the CD14- BDCA2- population, myeloid dendritic cells (mDCs) (CD123- BDCA1+) and a CD123 intermediate myeloid DC subset (CD123int BDCA1+) were identified. From the CD14- BDCA2+ population, plasmacytoid DCs (CD123hi, BDCA1-) were identified.

The granulocyte-rich red cell pellet from Ficoll density centrifugation was lysed twice in ammonium chloride lysis buffer (#7850, Stem Cell Technologies) to deplete red blood cells, prior to negative selection of eosinophils (untouched) using the eosinophil isolation kit (#130-050-010, Miltenyi Biotec). The remaining fraction of the cells contained the neutrophils. The cells purity was checked by flow cytometry: eosinophils (SSC hi, CD16lo) and neutrophils (SSC Hi, CD16hi).

Example FACS plots for the sorted populations can be found in Supplementary Figure 2. Purity checks were performed as possible, with 94 +/- 6 (sd) purity.

# RNA extraction

Isolated human cell subsets were stabilised in Qiazol (#79306, Qiagen) and frozen at -80°C until RNA extraction. RNA was isolated using the miRNeasy Kit (#217004, Qiagen) with on-column DNA removal using the RNase-free DNA removal kit (#79254, Qiagen).

Isolated mouse cell subsets were stabilized in RLT Buffer (#79216, Qiagen) and frozen at -80°C. Total RNA was isolated using the RNeasy Micro Kit (#74004, Qiagen).

RNA concentration and integrity was measured using the RNA 6000 Nano Kit (#5067-1512, Agilent Technologies) and the 2100 Bioanalyzer (Agilent Technologies).

**SUPPLEMENTARY DATA**

Supplementary Table 1: Haemopedia RNA-seq Mouse Immunophenotypes

Supplementary Table 2: Antibodies used in the Haemopedia RNA-seq cell collection

Supplementary Table 3: Haemopedia RNA-seq Human Immunophenotypes

Supplementary Table 4: Summary of microarray datasets included in the repository

Supplementary Figure 1: Flow cytometry sorting strategy for cell types included in the Mouse Haemopedia RNA-Seq.

Plots show gating strategy and sort profile for a sample included in the Haemopedia atlas.

Supplementary Figure 2: Flow cytometry sorting strategy for cell types included in the Human Haemopedia RNA-Seq.

Plots show gating strategy and sort profile for a sample included in the Haemopedia atlas.
