# Supplementary material for: Haemopedia RNA-seq: a database of gene expression during haematopoiesis in mice and humans
Source: Nucleic Acids Res. 2018 Nov 5;47(Database issue):D780–5. doi: 10.1093/nar/gky1020 (PMC6324085; doi:10.1093/nar/gky1020)
Supplement: Supplementary Data [file gky1020_supplemental_files.zip › Sup Fig1 MouseSortOutline.pdf]

Sort strategy

Mac and PtB

Direct stain with markers to remove myeloid cells

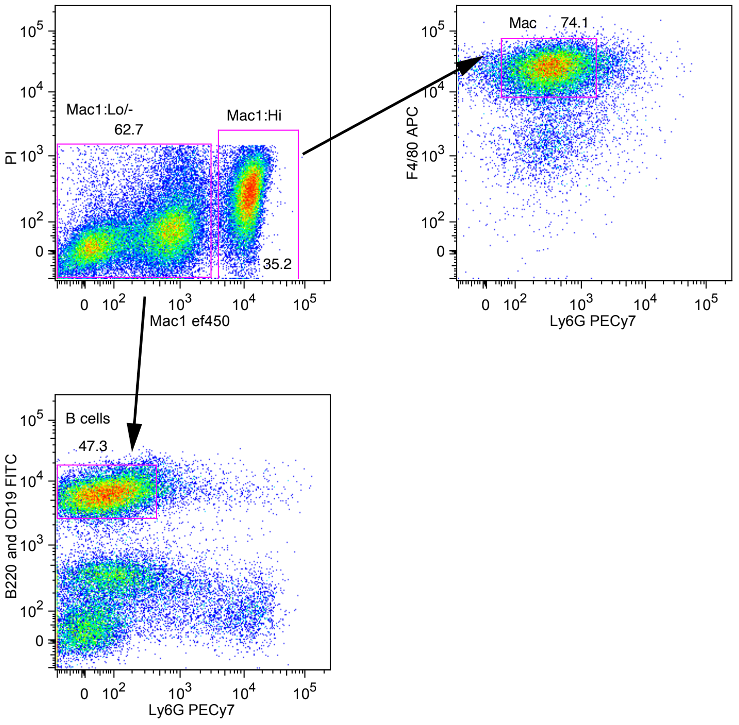

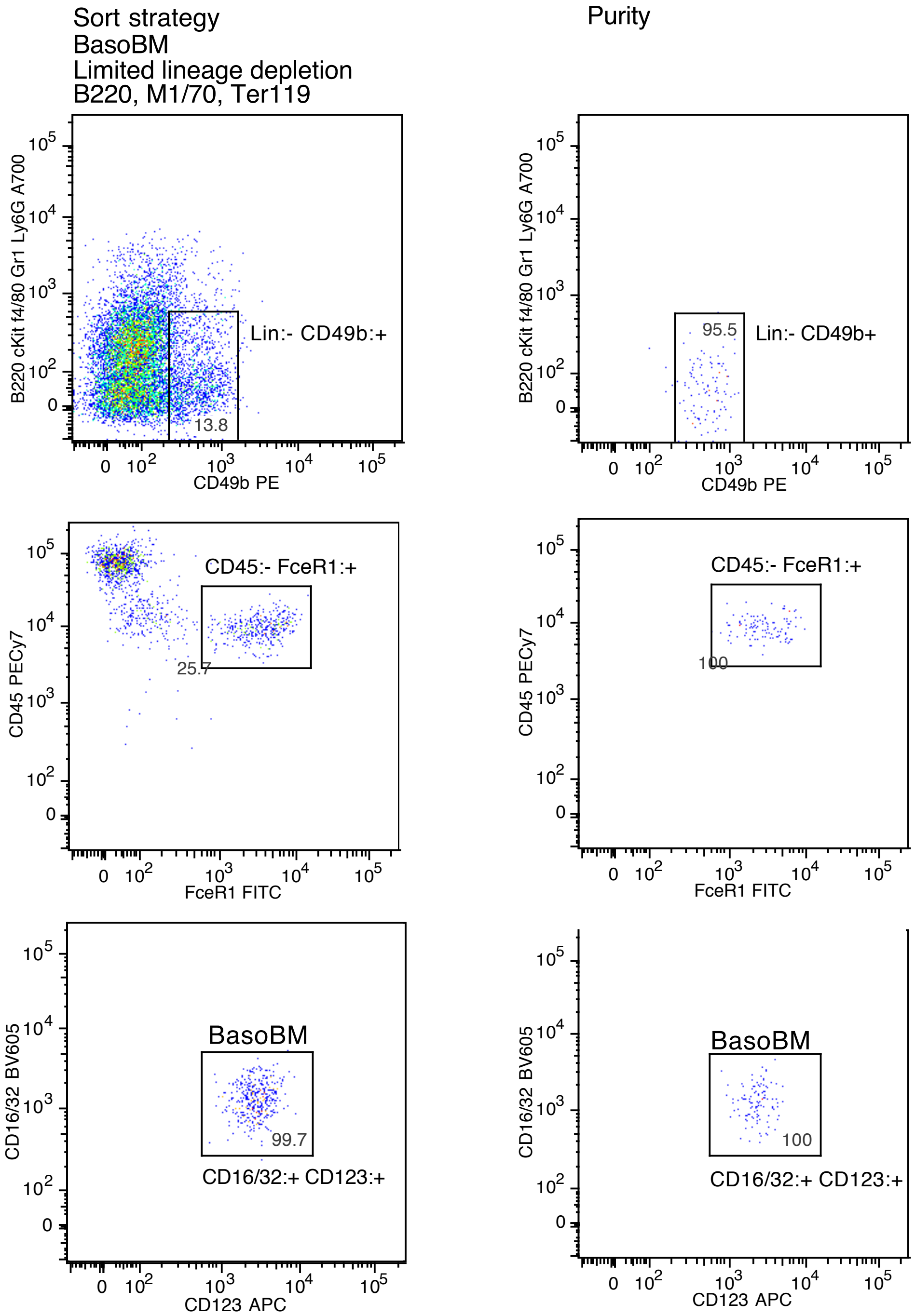

Sort strategy
BasoBM
Limited lineage depletion
B220, M1/70, Ter119
Purity
B220 cKit f4/80 Gr1 Ly6G A700
CD49b PE
Lin:- CD49b:+
13.8
95.5
Lin:- CD49b+
CD45 PECy7
FceR1 FITC
CD45:- FceR1:+
25.7
100
CD16/32 BV605
CD123 APC
BasoBM
99.7
100
CD16/32:+ CD123:+

Sort strategy
BasoCult
day 10 BM culture with SCF + IL3

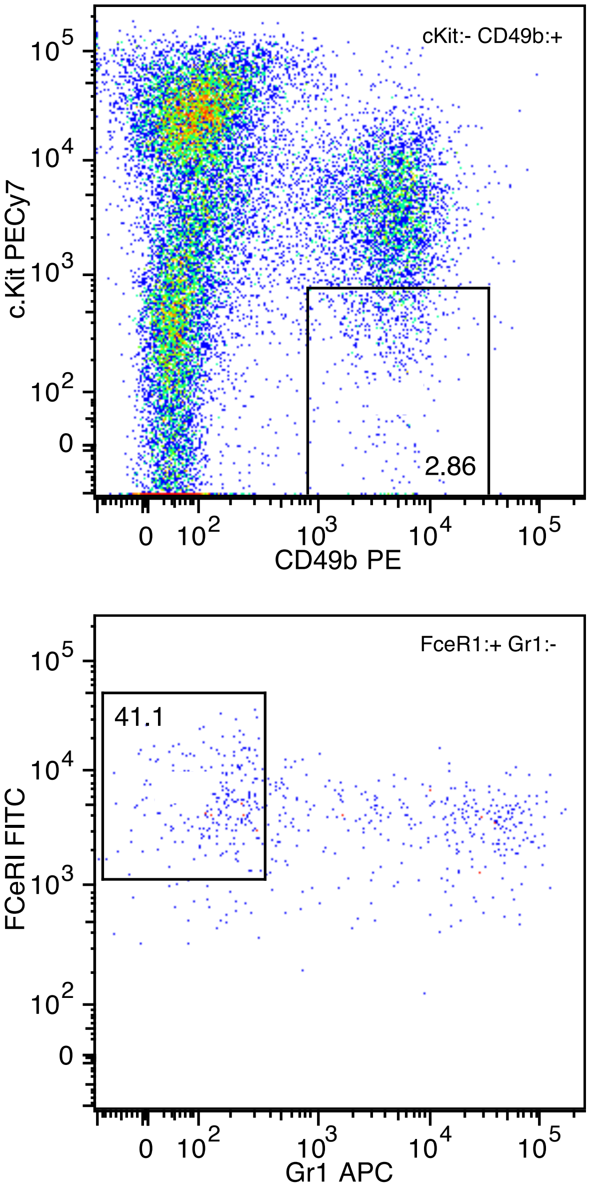

Sort strategy
BasoSpl
Limited lineage depletion
B220, M1/70, Ter119

Purity

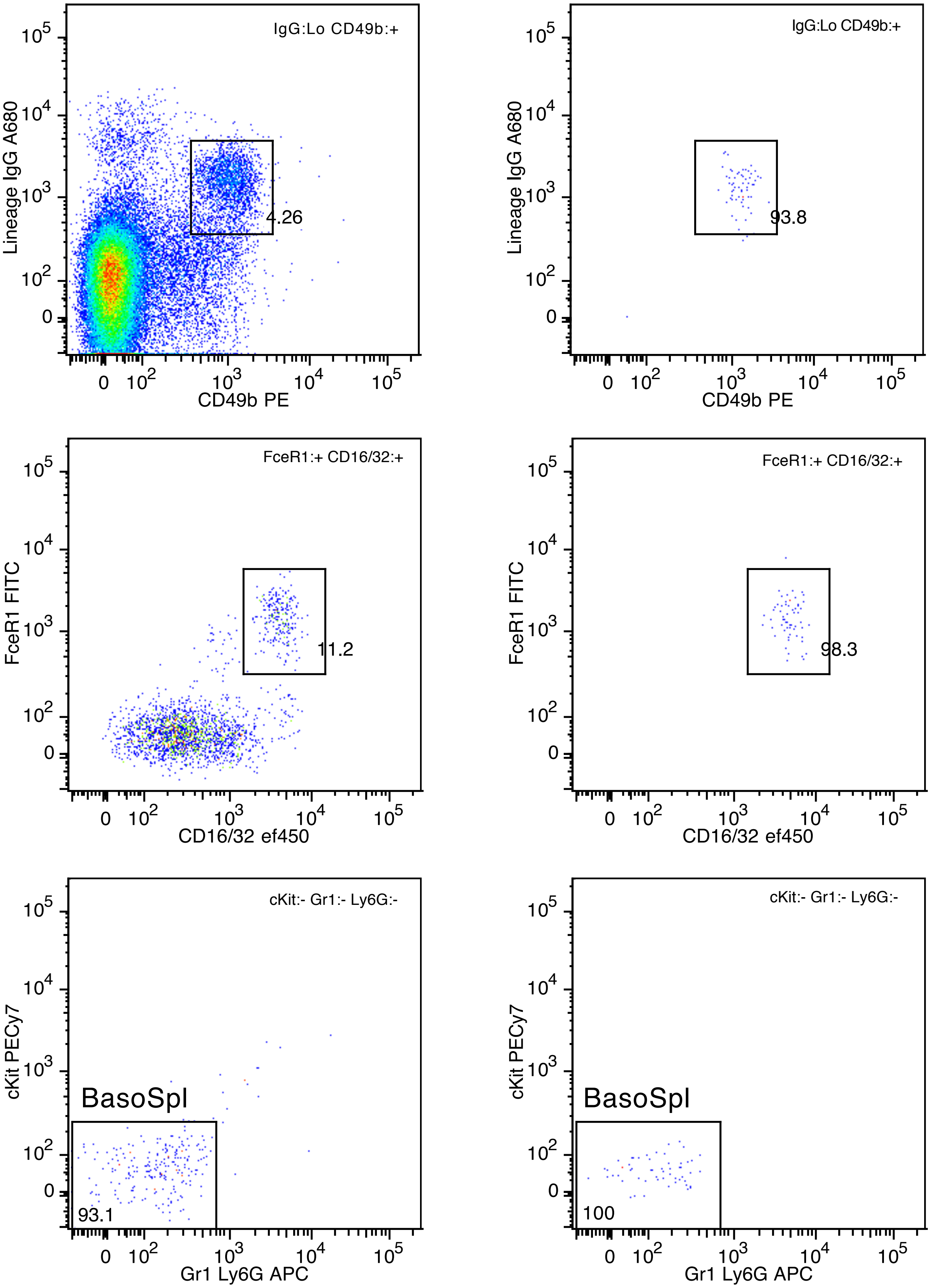

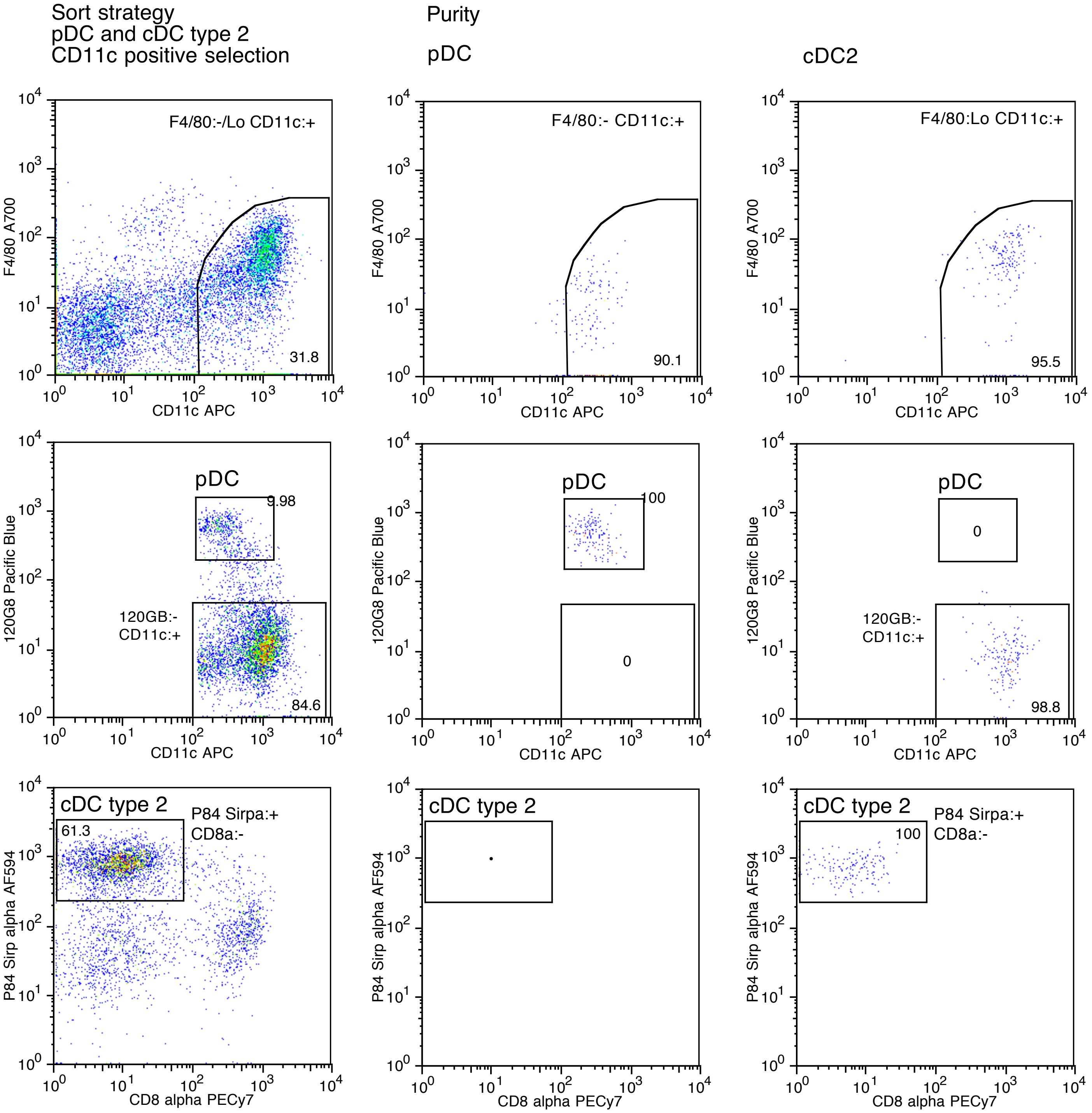

Sort strategy
pDC and cDC type 2
CD11c positive selection
Purity
pDC
cDC2
F4/80:-/Lo CD11c:+
31.8
F4/80:- CD11c:+
90.1
F4/80:Lo CD11c:+
95.5
F4/80 A700
CD11c APC
pDC
9.98
120GB:-
CD11c:+
84.6
100
0
98.8
120G8 Pacific Blue
cDC type 2
61.3
P84 Sirpa:+
CD8a:-
100
P84 Sirp alpha AF594
CD8 alpha PECy7

Sort strategy

Eo

Lineage depletion B220, CD3, CD4 CD8, CD19, Ter119

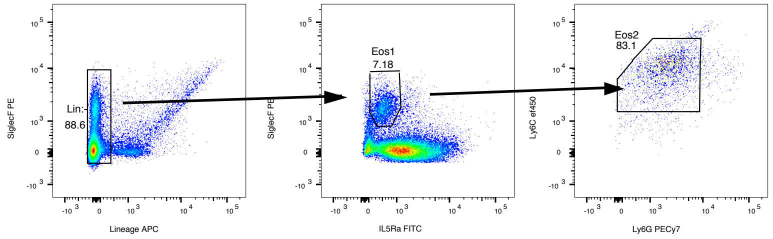

Purity

Eo

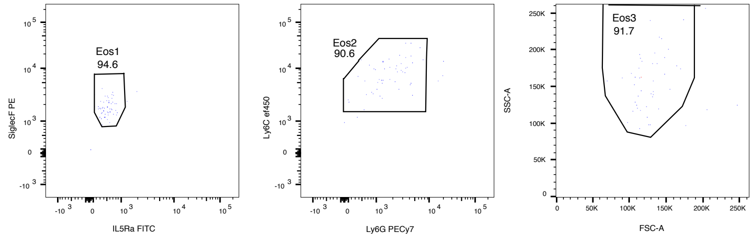

Sort strategy
Cultured Eos

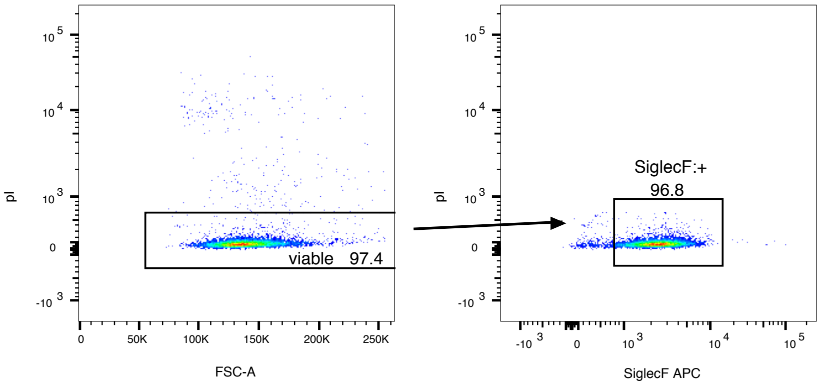

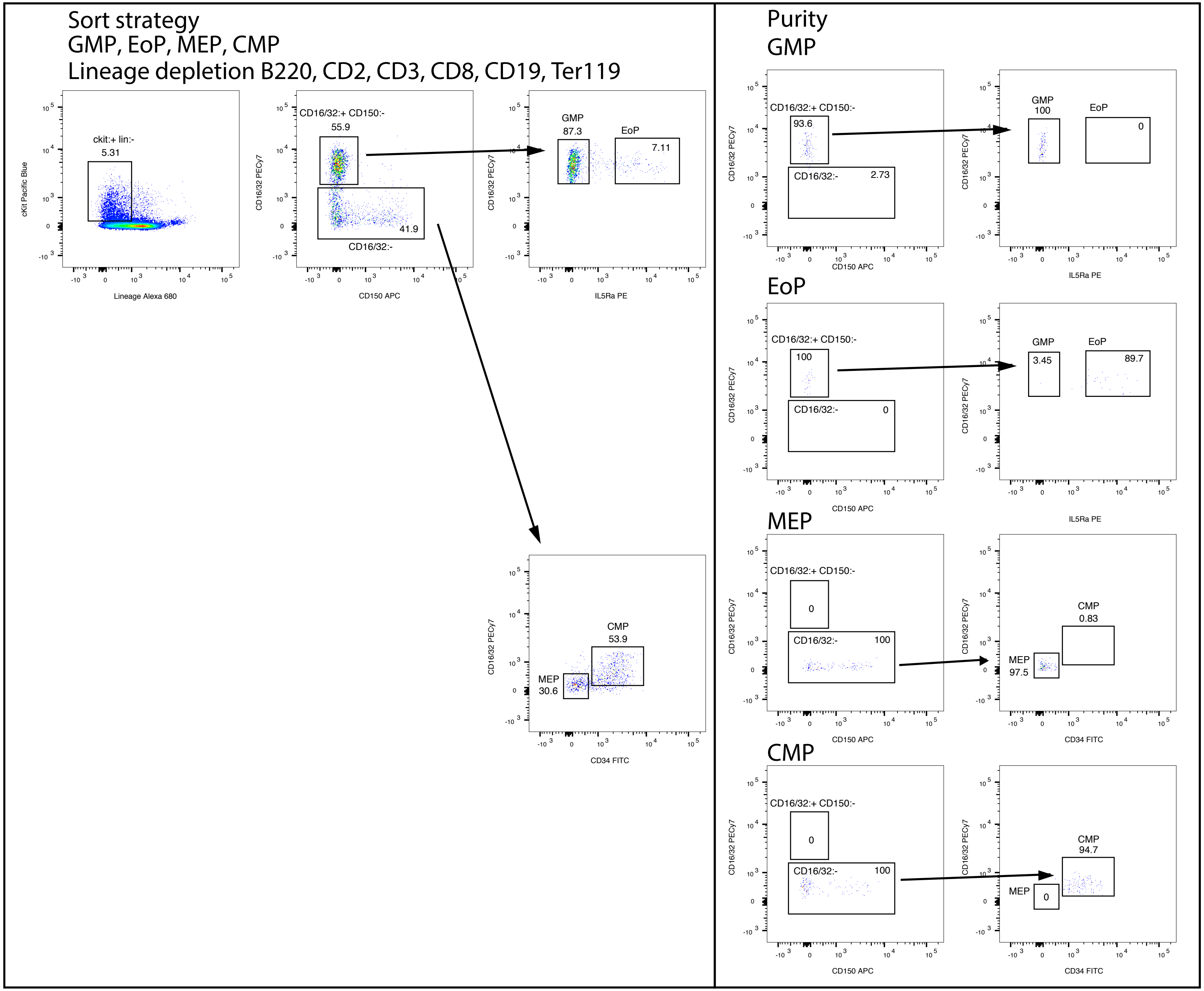
Sort strategy
GMP, EoP, MEP, CMP
Lineage depletion B220, CD2, CD3, CD8, CD19, Ter119
ckit:+ lin:-
5.31
cKit Pacific Blue
Lineage Alexa 680
CD16/32:+ CD150:-
55.9
41.9
CD16/32:-
CD16/32 PECy7
CD150 APC
GMP
87.3
EoP
7.11
IL5Ra PE
CMP
53.9
MEP
30.6
CD34 FITC
Purity
GMP
93.6
2.73
100
0
EoP
3.45
89.7
MEP
0.83
97.5
CMP
94.7

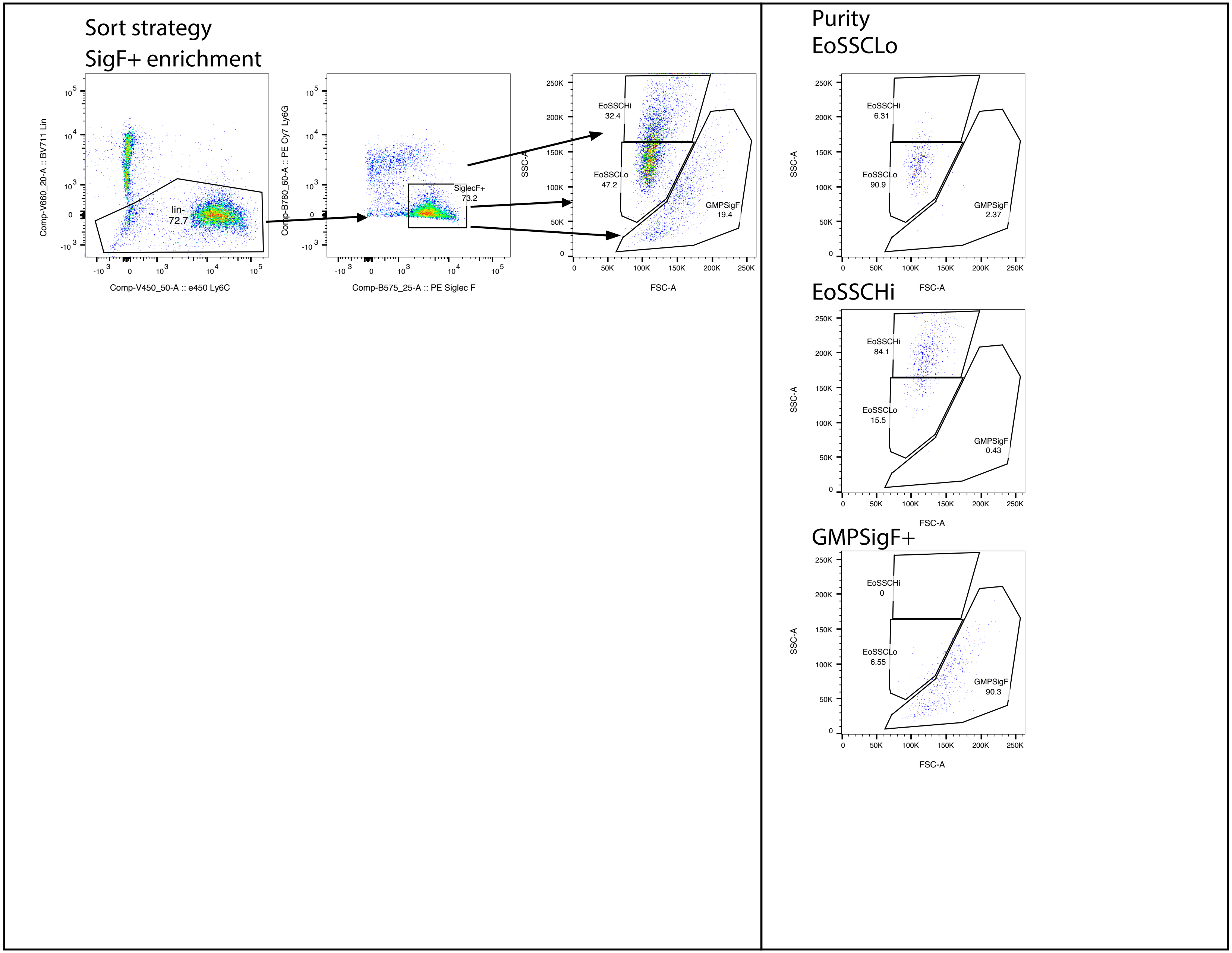
Sort strategy
SigF+ enrichment
Comp-V660_20-A :: BV711 Lin
Comp-V450_50-A :: e450 Ly6C
lin-
72.7
Comp-B780_60-A :: PE Cy7 Ly6G
Comp-B575_25-A :: PE Siglec F
SiglecF+
73.2
SSC-A
FSC-A
EoSSCHi
32.4
EoSSCLo
47.2
GMPSigF
19.4
Purity
EoSSCLo
EoSSCHi
6.31
EoSSCLo
90.9
GMPSigF
2.37
EoSSCHi
EoSSCHi
84.1
EoSSCLo
15.5
GMPSigF
0.43
GMPSigF+
EoSSCHi
0
EoSSCLo
6.55
GMPSigF
90.3

Lineage negative

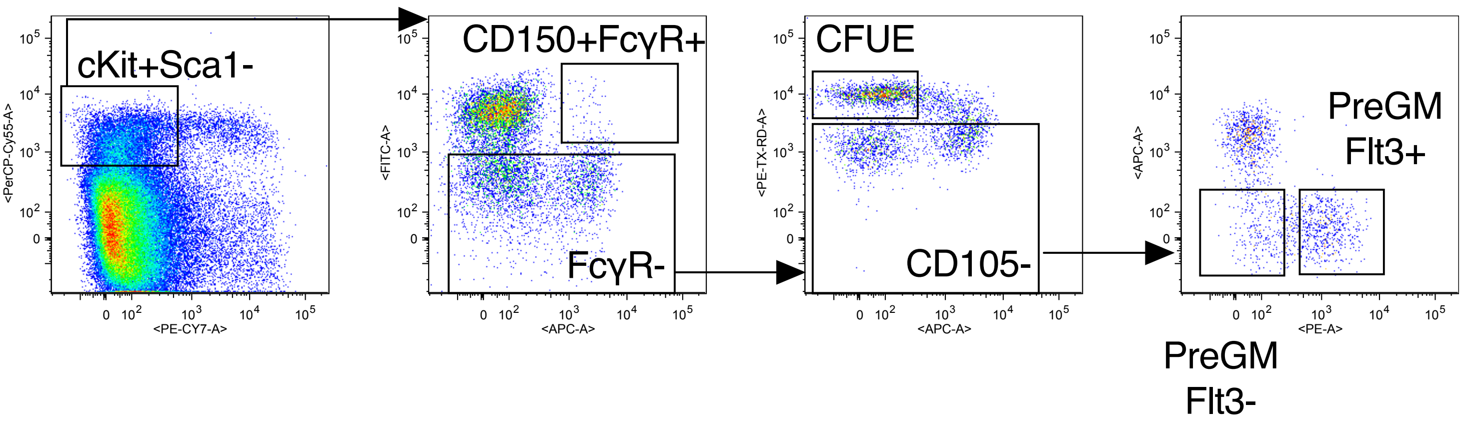

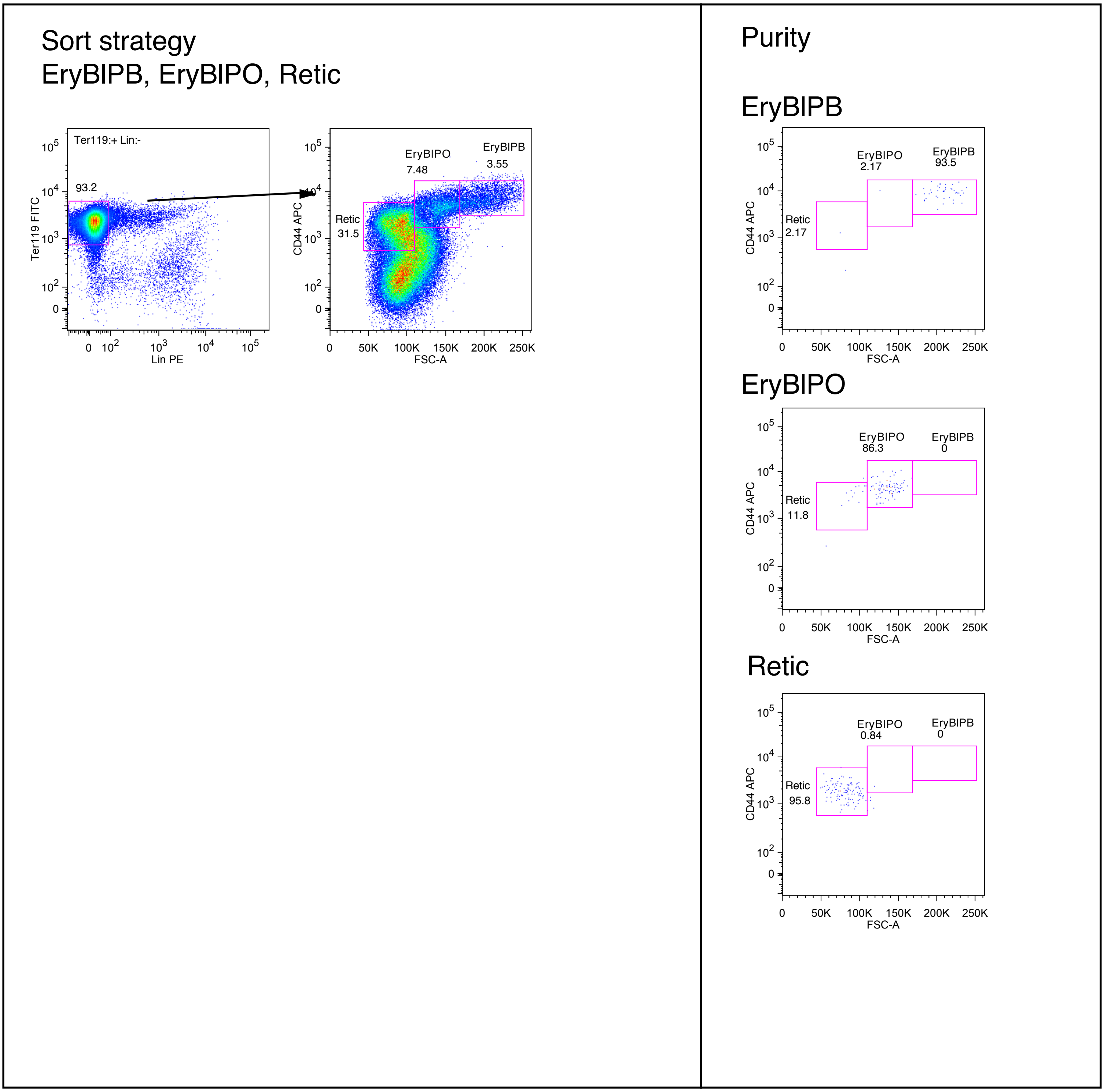

Sort strategy
EryBlPB, EryBlPO, Retic
Ter119:+ Lin:-
93.2
Ter119 FITC
Lin PE
EryBlPO
7.48
EryBlPB
3.55
Retic
31.5
CD44 APC
FSC-A
Purity
EryBlPB
EryBlPO
2.17
EryBlPB
93.5
Retic
2.17
EryBlPO
EryBlPO
86.3
EryBlPB
0
Retic
11.8
Retic
EryBlPO
0.84
EryBlPB
0
Retic
95.8

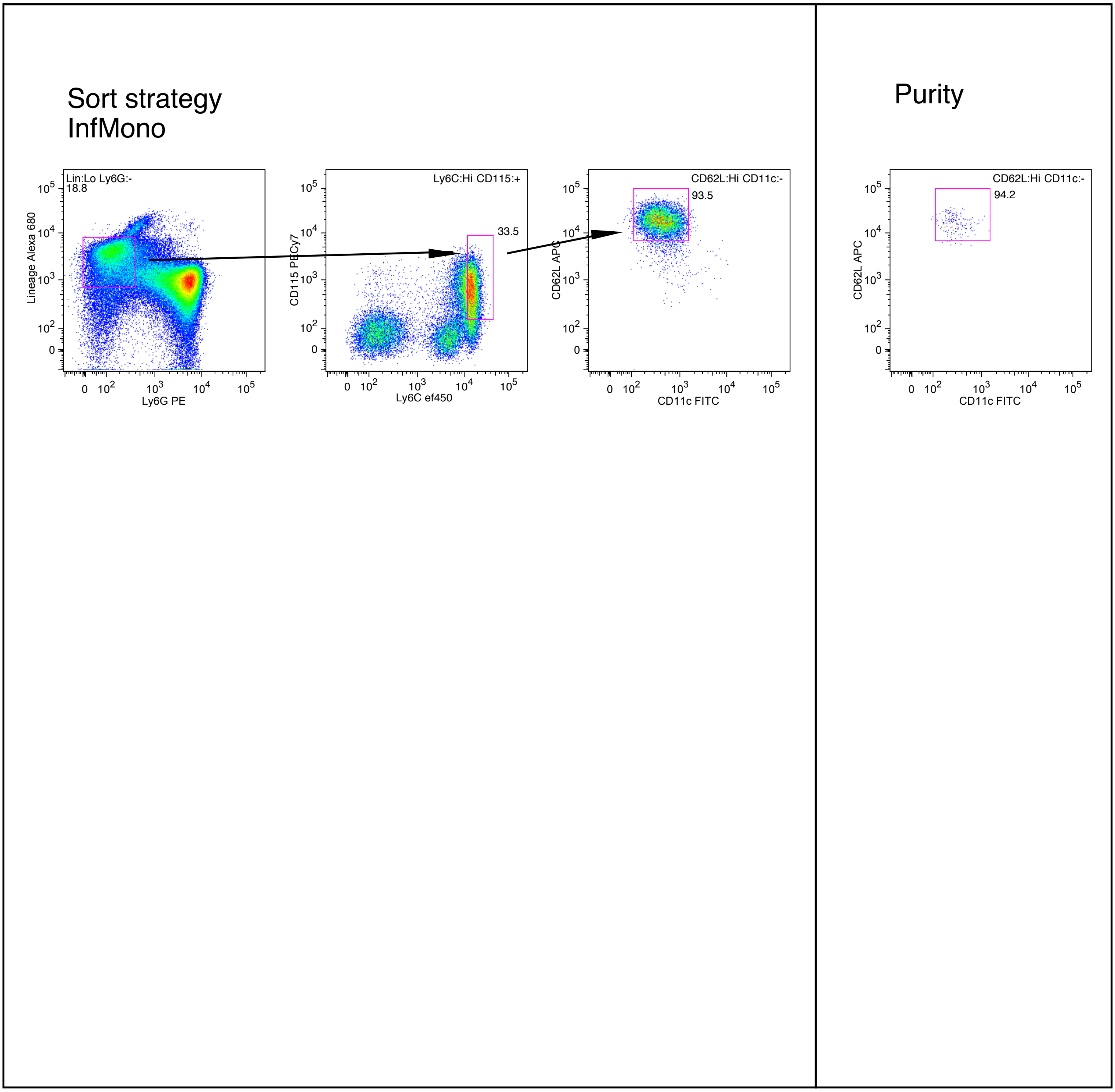

Sort strategy
InfMono
Purity
Lin:Lo Ly6G:-
18.8
Lineage Alexa 680
Ly6G PE
Ly6C:Hi CD115:+
33.5
CD115 PECy7
Ly6C ef450
CD62L:Hi CD11c:-
93.5
CD62L APC
CD11c FITC
CD62L:Hi CD11c:-
94.2
CD62L APC
CD11c FITC

Sort strategy
Direct stain
MacCult

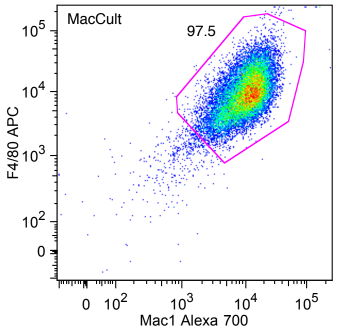

Purity

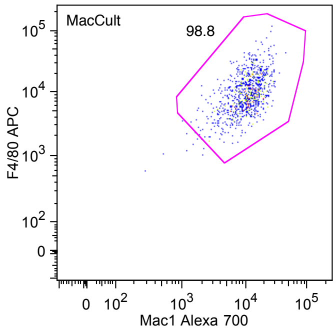

Sort strategy

MonoBM

Limited lineage B220, CD2, CD3, CD8, CD19, Ter119

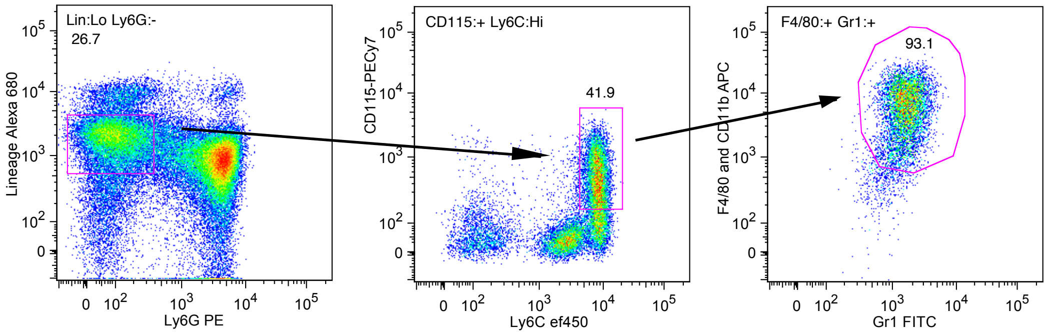

Purity

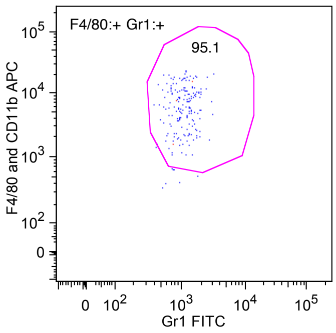

Sort strategy
MonoPB

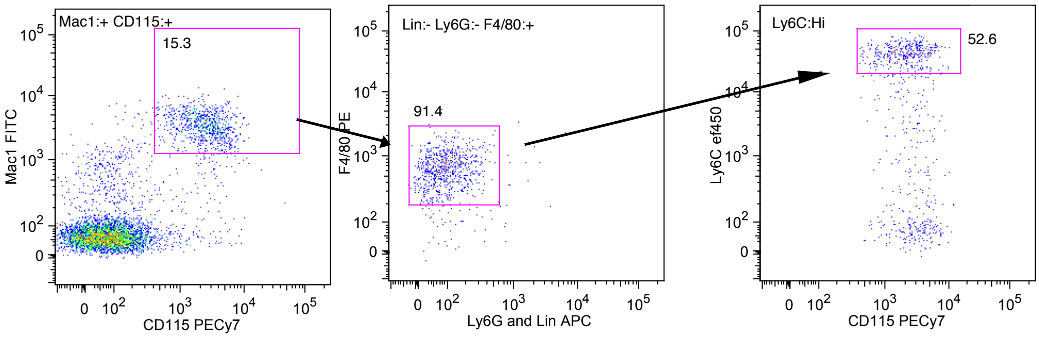

Purity

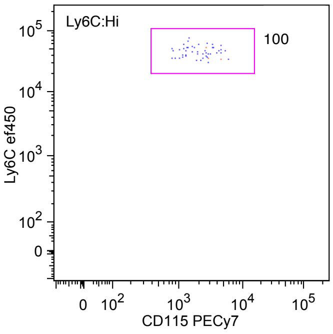

Sort strategy

Mast

Direct stain with B cell and erythrocyte exclusion

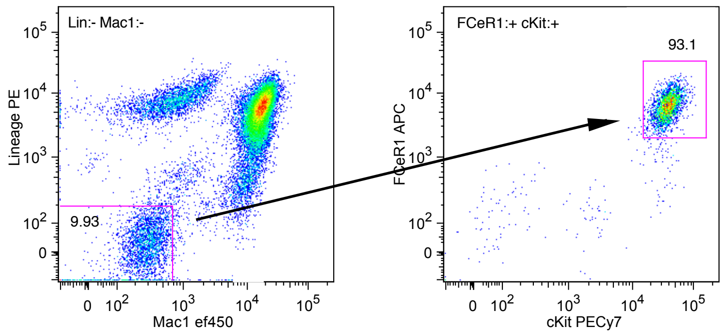

Purity

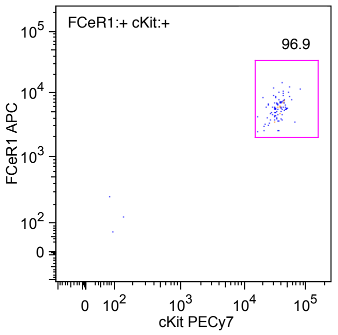

Purity check of cultured megakaryocytes (MegTPO)

Ploidy Profile: 45% 32N or bigger

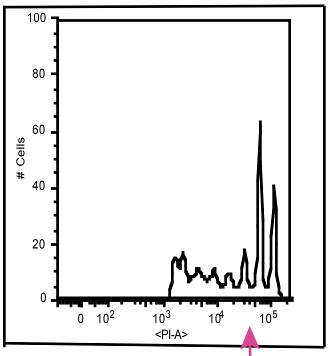

32N

Cytospin

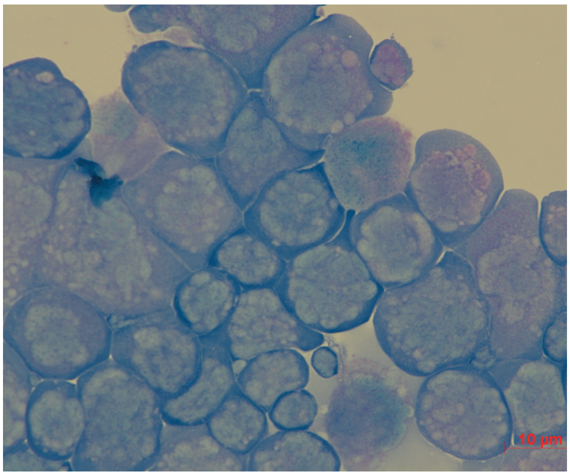

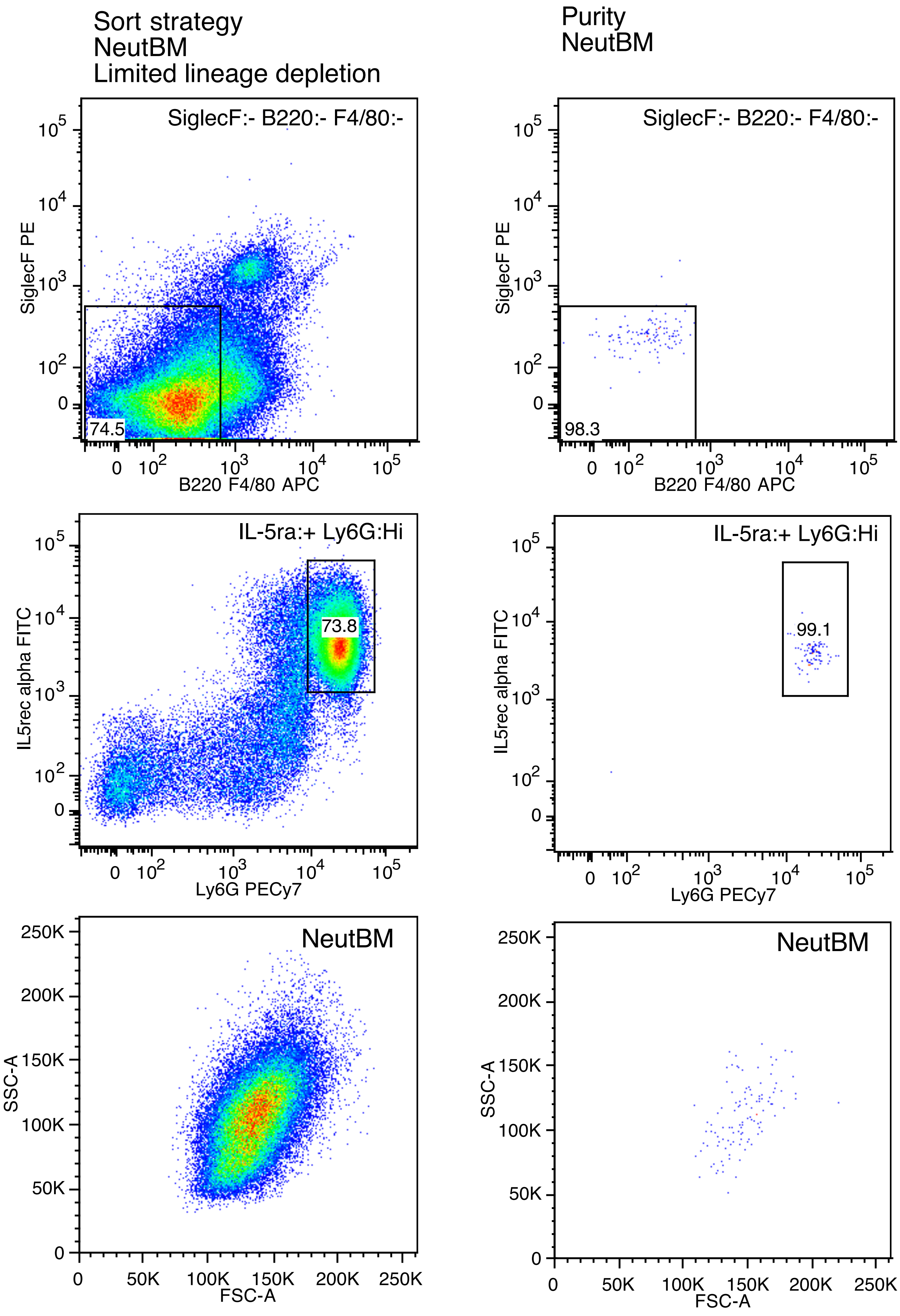
Sort strategy
NeutBM
Limited lineage depletion
Purity
NeutBM
SiglecF:- B220:- F4/80:-
SiglecF PE
B220 F4/80 APC
74.5
98.3
IL-5ra:+ Ly6G:Hi
IL5rec alpha FITC
Ly6G PECy7
73.8
99.1
NeutBM
SSC-A
FSC-A
0
50K
100K
150K
200K
250K

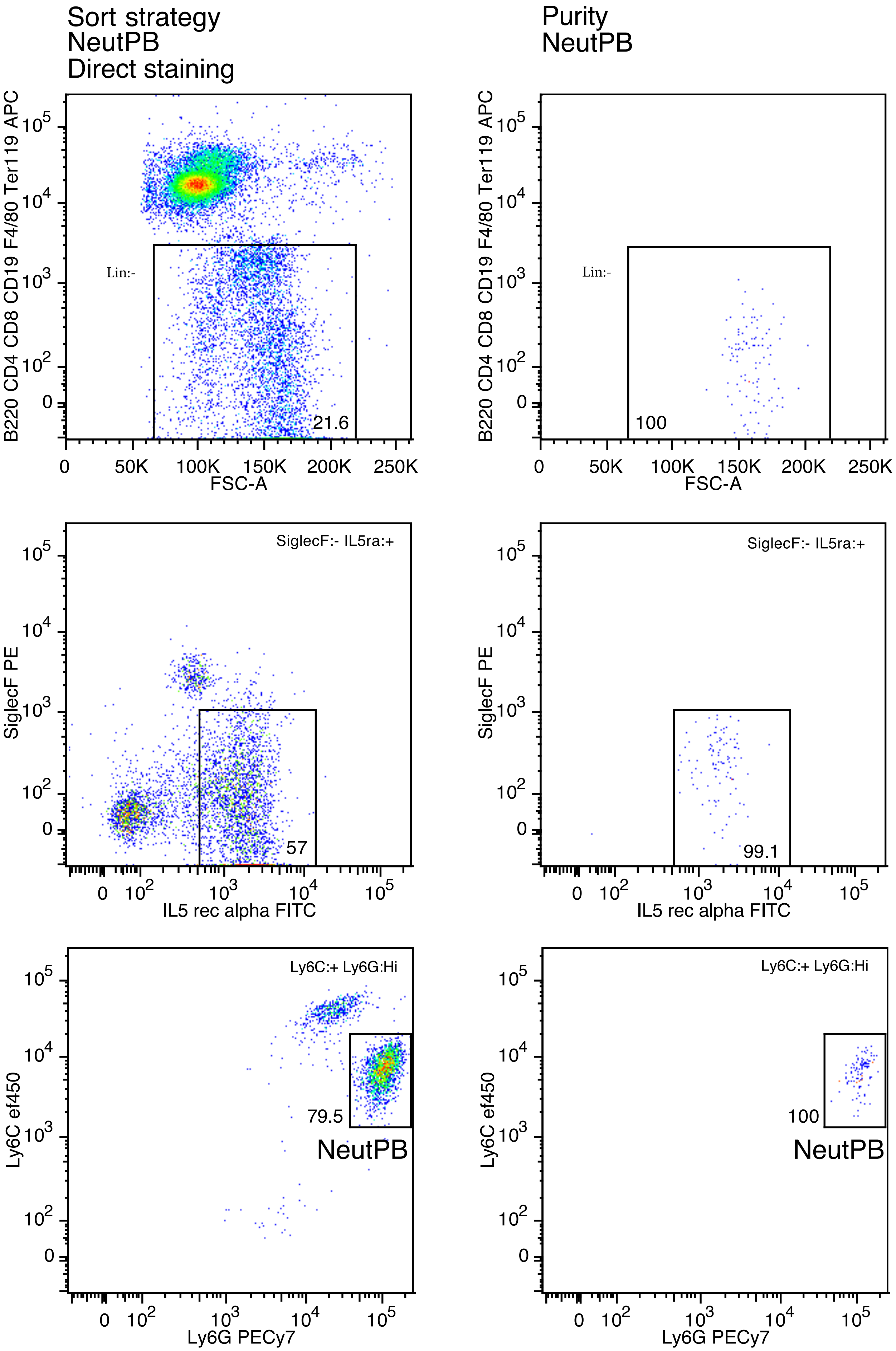

Sort strategy
NeutPB
Direct staining
Purity
NeutPB
B220 CD4 CD8 CD19 F4/80 Ter119 APC
FSC-A
Lin:-
21.6
100
SiglecF PE
IL5 rec alpha FITC
SiglecF:- IL5ra:+
57
99.1
Ly6C ef450
Ly6G PECy7
Ly6C:+ Ly6G:Hi
79.5
100
NeutPB

Sort strategy
NK
Lineage depletion
B220, CD3, CD4, CD8, CD19 Gr1 Ter119

Purity
NK

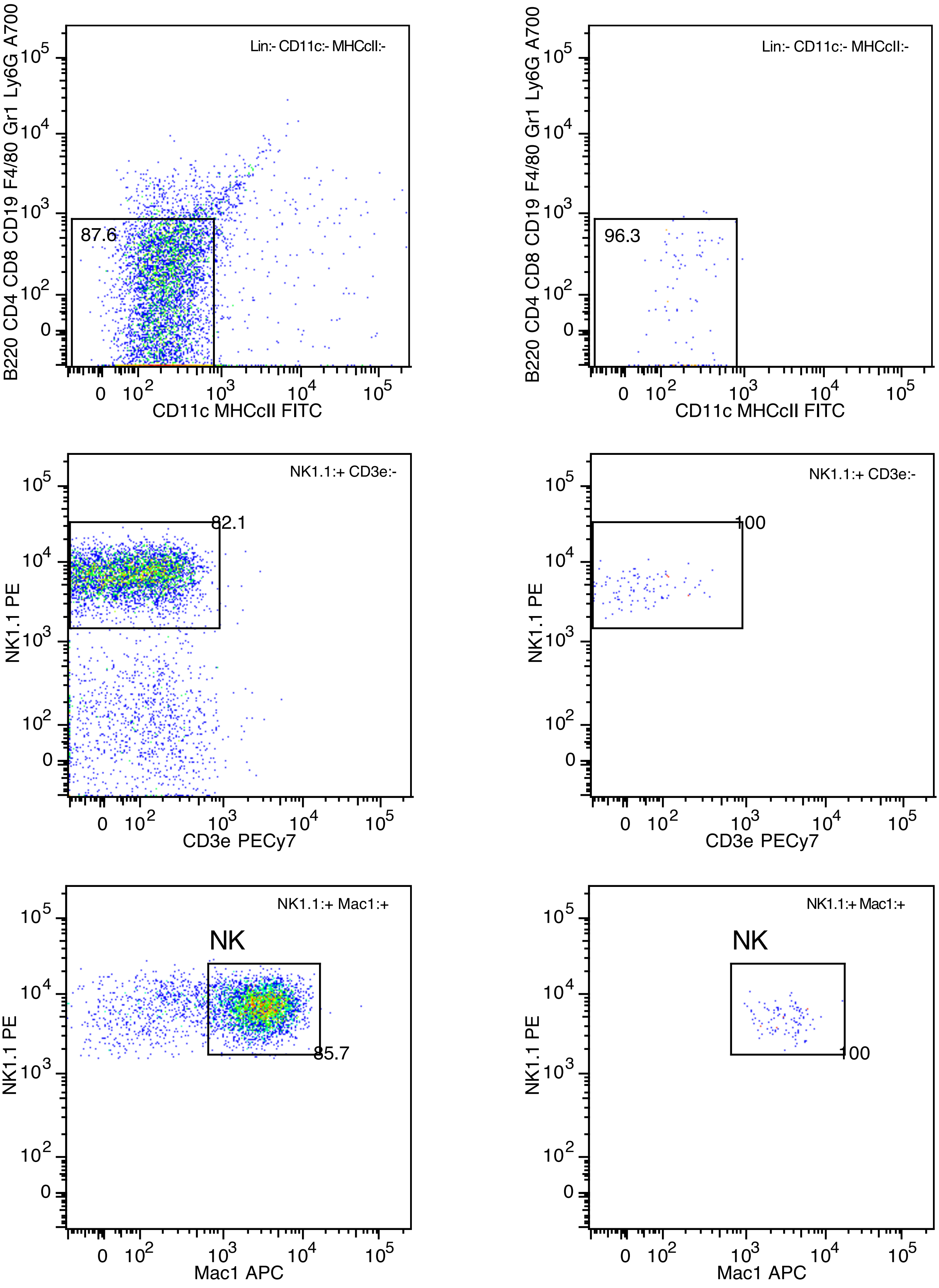

GMP_IRF8Lo Int Hi

Sort Strategy

Lineage depletion: Ter119, B220, Gr1 (1A8), Mac1 (M1/70), CD4, CD8

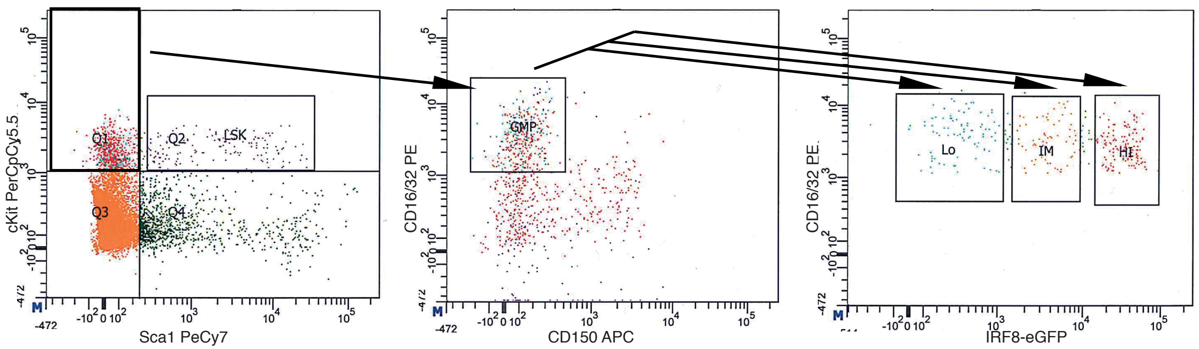

Sort strategy

CD4T

Lineage depletion B220, CD8, CD19, Gr1, M1/70, Ter119

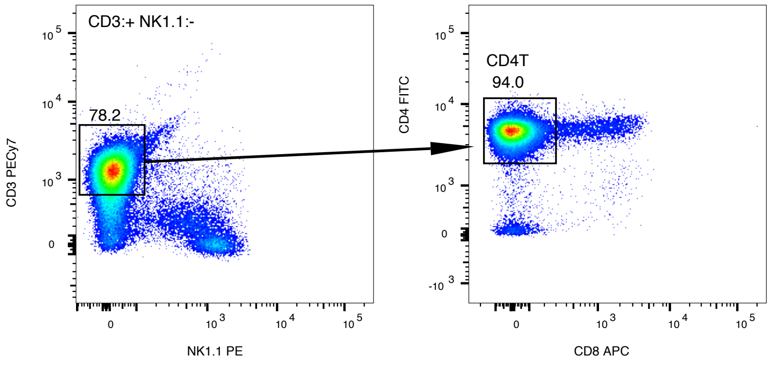

Purity

CD4T

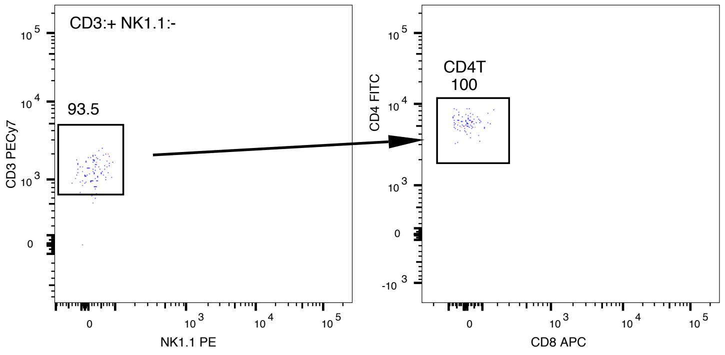

Sort strategy

CD8T

Lineage depletion B220, CD4, CD19, Gr1, M1/70, Ter119

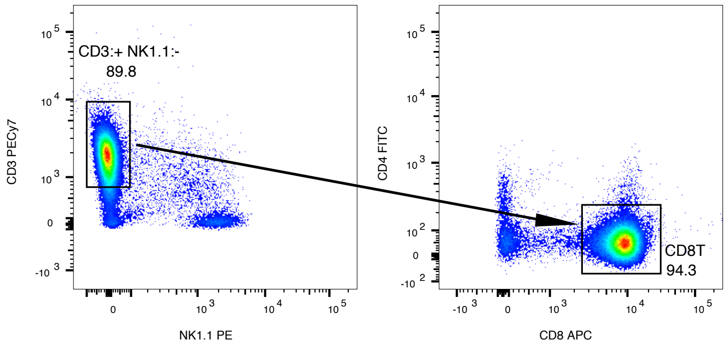

Purity

CD8T

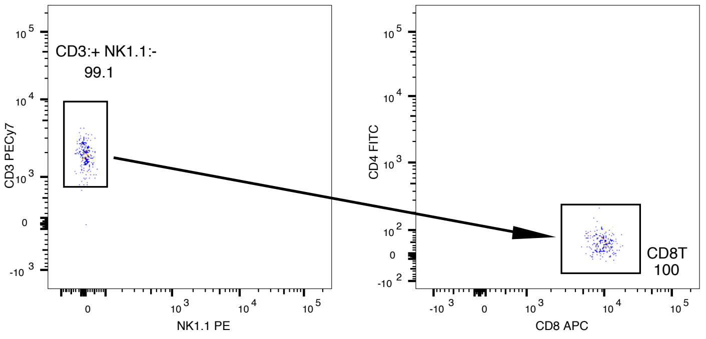

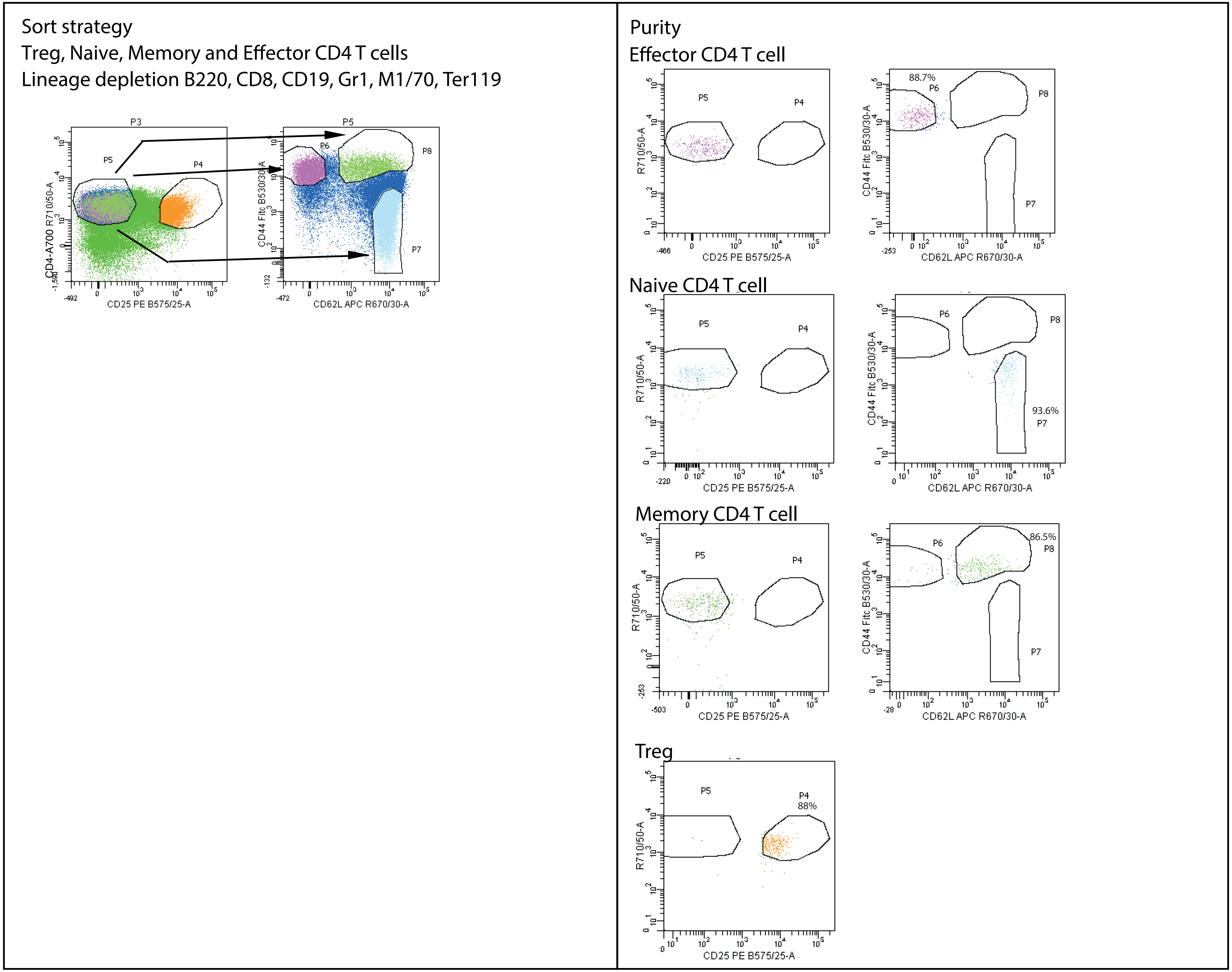
Sort strategy
Treg, Naive, Memory and Effector CD4 T cells
Lineage depletion B220, CD8, CD19, Gr1, M1/70, Ter119
P3
P5
P4
P6
P8
P7
CD4-A700 R710/50-A
CD25 PE B575/25-A
CD44 Fitc B530/30-A
CD62L APC R670/30-A
Purity
Effector CD4 T cell
88.7%
Naive CD4 T cell
93.6%
Memory CD4 T cell
86.5%
Treg
88%
R710/50-A

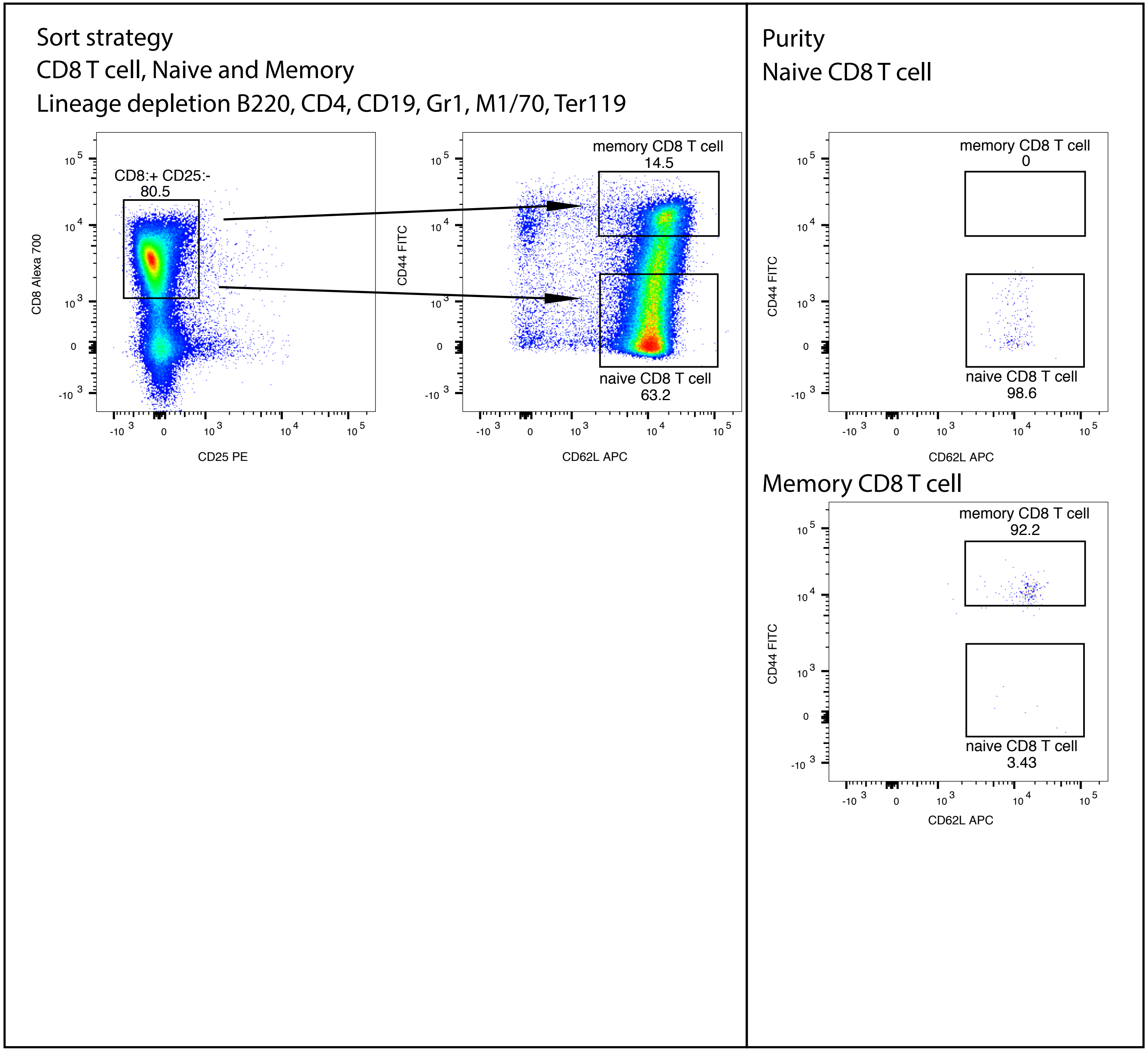

Sort strategy
CD8 T cell, Naive and Memory
Lineage depletion B220, CD4, CD19, Gr1, M1/70, Ter119
CD8:+ CD25:-
80.5
CD8 Alexa 700
CD25 PE
memory CD8 T cell
14.5
naive CD8 T cell
63.2
CD44 FITC
CD62L APC
Purity
Naive CD8 T cell
memory CD8 T cell
0
naive CD8 T cell
98.6
CD44 FITC
CD62L APC
Memory CD8 T cell
memory CD8 T cell
92.2
naive CD8 T cell
3.43
CD44 FITC
CD62L APC
