# Supplementary figures and images for: Haemopedia RNA-seq: a database of gene expression during haematopoiesis in mice and humans
Source: Nucleic Acids Res. 2018 Nov 5;47(Database issue):D780–5. doi: 10.1093/nar/gky1020 (PMC6324085; doi:10.1093/nar/gky1020)

Sort Strategy: Lymphocytes

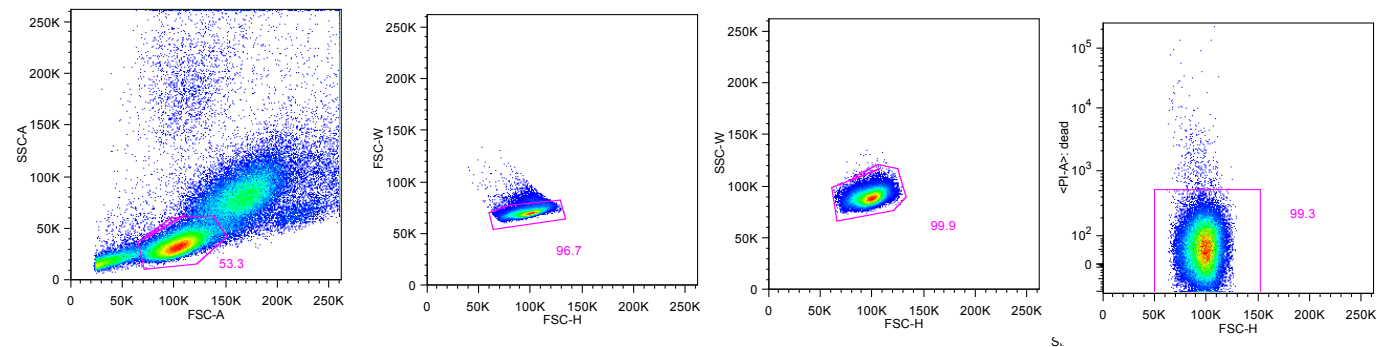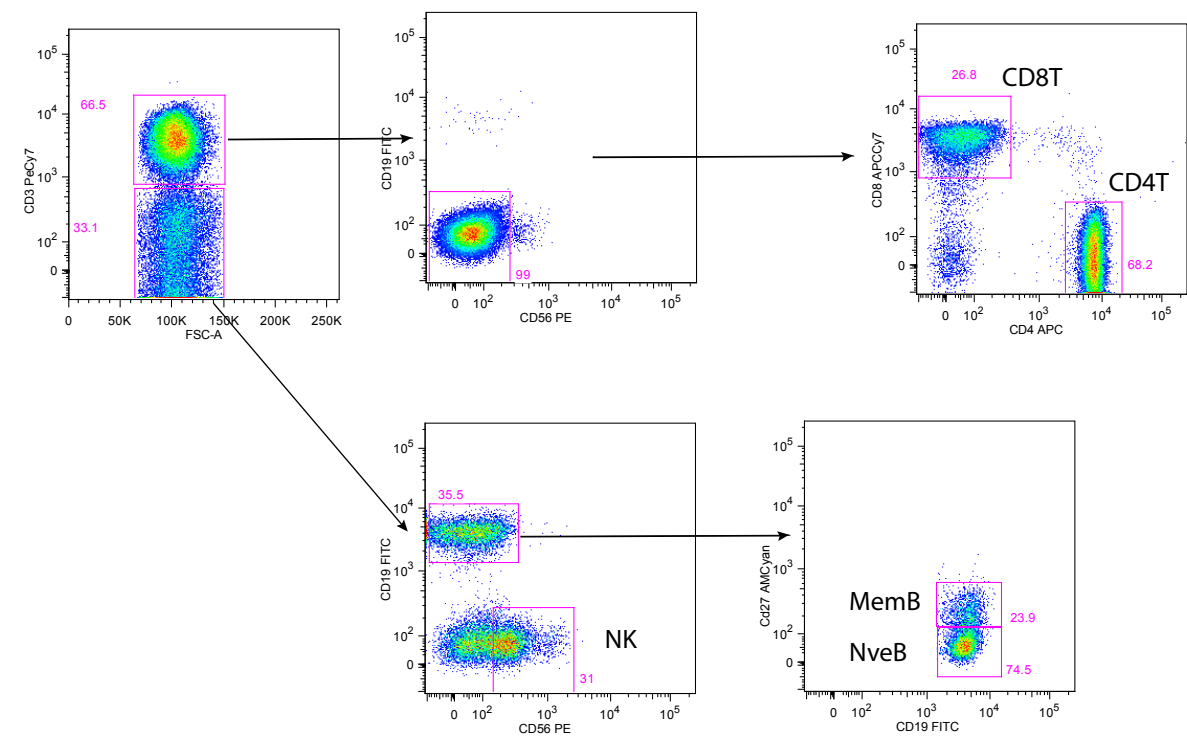

## Sort Strategy: Monocytes and Dendritic Cells

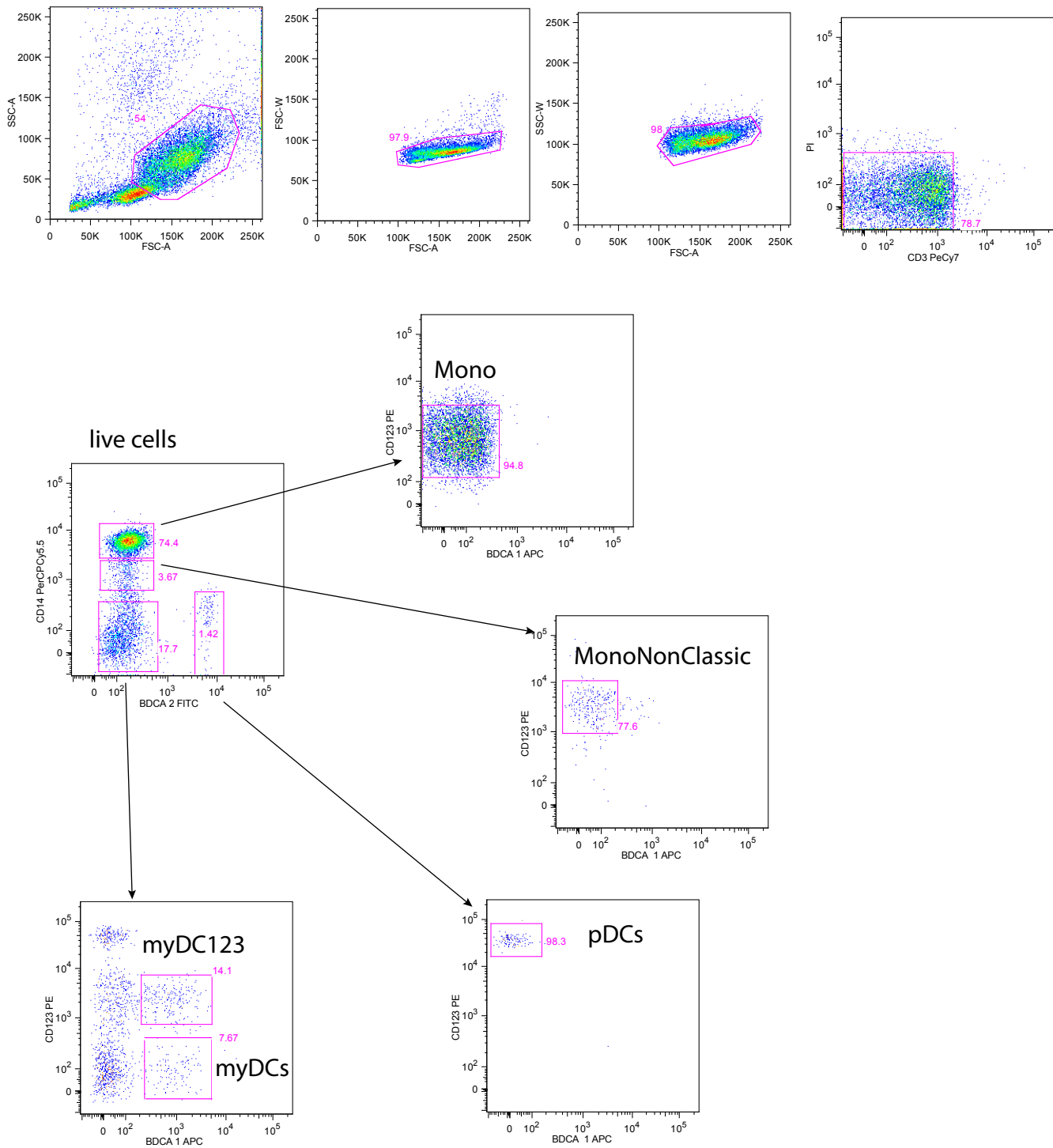

Supplement: Supplementary Data [file gky1020_supplemental_files.zip › Sup Fig2 HumanSortStrategy.pdf]
